# Ultrasoft Yet Tough Multifunctional Organohydrogels Enabled by Molecular Chain Lubrication Strategy for Self‐Powered Wearable Electronics

**DOI:** 10.1002/advs.202522686

**Published:** 2026-01-30

**Authors:** Si Wang, Lin Hu, Mingbo Pu, Mingfeng Xu, Tao Zhou, Hanbing Rao, Xiong Li, Xiaoliang Ma, Gehong Su, Xiangang Luo

**Affiliations:** ^1^ State Key Laboratory of Optical Field Manipulation Science and Technology Institute of Optics and Electronics Chinese Academy of Sciences Chengdu China; ^2^ College of Science Sichuan Agricultural University Ya'an China; ^3^ College of Materials Sciences and Opto‐Electronic Technology University of Chinese Academy of Sciences Beijing China; ^4^ Research Center on Vector Optical Fields Institute of Optics and Electronics Chinese Academy of Sciences Chengdu China; ^5^ State Key Laboratory of Polymer Materials Engineering of China Polymer Research Institute Sichuan University Chengdu China

**Keywords:** molecular chain lubrication, multifunctional, rganohydrogel, soft yet tough, triboelectric nanogenerator

## Abstract

Flexible triboelectric nanogenerators (TENGs) underscore the promise of skin‑inspired hydrogels as intelligent materials for wearable electronics. However, overcoming the intrinsic trade‑off between stiffness and toughness while integrating multifunctionality into the hydrogels remains a great challenge. Here, we propose a multifunctional supramolecular organohydrogel (AQGL) via a molecular chain lubrication strategy, where glycerol (Gly)/water clusters act as dynamic lubricants to modulate polymer interactions and cross‑linking structure within the HAPAA/QCS/LiCl (AQL) gel network. This regulation endows the AQGL with remarkable mechanical characteristics, yielding an ultrahigh stretchability (5800%), ultralow Young's modulus (14.3 kPa), high toughness (11.77 MJ/m^3^), and impressive fracture energy (75.96 kJ/m^2^). Meanwhile, the AQGL further demonstrates multifunctionality including excellent optical transparency (∼95%), exceptional environmental durability against drying/freezing (−40°C), notable anti‐icing capability, distinguished antibacterial activity, high conductivity, and good self‐healing property. When employed as a TENG electrode, its intrinsic self‐adhesiveness ensures strong interfacial stability with triboelectric layer, enabling excellent electrical output and reliable performance in motion monitoring, handwriting recognition, and autonomous energy harvesting. This study provides a rational design strategy toward soft yet tough, and multifunctional organohydrogels, advancing the development of self‑powered epidermal electronics.

## Graphical abstract

An ultrasoft yet tough organohydrogel (AQGL) with 5800% stretchability and 14.3 kPa modulus is developed via a novel molecular chain lubrication strategy. It features optical transparency, drying/freezing (−40°C) tolerance, antibacterial activity, conductivity, self‐healing, and adhesiveness. This multifunctional electrode enables high‐performance TENGs for wearable motion tracking, handwriting recognition, and autonomous energy harvesting.

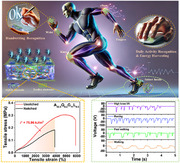

## 1 Introduction

The ongoing advancement in flexible electronics increasingly draws inspiration from the human skin owing to its unique flexibility, stretchability, and self‐healing capabilities, which collectively drive the integration of compliant materials into next‐generation wearable technologies [1, 2]. Nevertheless, conventional technologies are still hindered by limited autonomous power supply and poor adaptability to dynamic and complex environments, underscoring the urgent need for soft energy harvesting systems [3]. In this context, flexible triboelectric nanogenerators (TENGs) propose an effective route to efficiently convert ubiquitous mechanical energy into electricity for wearable electronics, offering a sustainable alternative to conventional energy systems plagued by intrinsic rigidity, short service life, frequent charging requirements, and environmental issues [4, 5, 6]. Hydrogels, with their intrinsic softness, stretchability, and tissue‐like compliance, provide an attractive platform for developing intelligent triboelectric skins applicable to robotics, human–machine interfaces (HMIs), and healthcare monitoring [7, 8].

Recent advances in hydrogel‑based TENGs have mainly focused on enhancing electrode stability and functional reliability through diverse material strategies. To enhance electrical performance, strategies such as incorporating conductive nanofillers (e.g., polyaniline, MXene, carbon nanotubes) or engineering ionically conductive networks have yielded encouraging progress [9, 10]. Especially, ionically conductive hydrogels provide promising stretchable electrodes for self‐powered electronics by integrating deformable polymer networks like polyvinyl alcohol (PVA), poly(acrylic acid) (PAA), chitosan, polyacrylamide (PAM) or polyvinyl pyrrolidone (PVP) with highly mobile ionic carriers (e.g., Li^+^, Na^+^, Cl^−^), offering uniform charge distribution, improved interfacial conformity, and sustained conductivity [11, 12]. Meanwhile, prompting exploration of self‐adhesive property has been devoted to strengthen the interfacial contact with triboelectric layers, which is beneficial to prompt the output performances [13, 14]. Despite such advances, conventional single‐network hydrogels remain intrinsically fragile with undesirable fracture energies typically below 10 J/m^2^, which are orders of magnitude lower than biological tissues (e.g., ∼1 kJ/m^2^ for cartilage, ∼2.5 kJ/m^2^ for skeletal muscle, ∼30 kJ/m^2^ for tendon) [15]. However, according to the Lake–Thomas model, there exists an intrinsic trade‑off between stiffness and toughness [16, 17]. Thus, designing and developing reinforced hydrogels without enhancing the modulus while achieving a synergy of mechanical property and environmental adaptability remains challenging in the development of synthetic materials [15, 18].

As for conventional toughening strategies, enhancing energy dissipation is often accompanied by an increase in Young's modulus, which leads to an inherent conflict with the compliant modulus range (tens to a few hundred kPa) required for conformal integration with dynamic soft tissues such as human skin [19]. For instance, toughening strategies such as double‐network (DN) approach [14, 20], ionic cross‐linking [21, 22], Hofmeister effect [23, 24], solvent exchange [25, 26], molecular chain entanglement [27, 28], and freeze‐casting techniques [29, 30] have been explored to enhance the mechanical properties of the hydrogels. In general, the DN approach enhances energy dissipation via introducing a secondary polymer network, but simultaneously raises solid content and cross‐link density, resulting in increased Young's modulus. Similarly, methods involving the Hofmeister effect or solvent exchange can result in volume shrinkage of the hydrogel, enhancing its toughness but also inadvertently increasing stiffness [16]. To develop strong and tough electrode for self‐powered applications, Peng et al. reported an ion‐gel with enhanced toughness of 22 MJ/m^3^ via solvent replacement and ion hybridization, yet the high elastic modulus (30 MPa) significantly reduced the softness and deformability required for wearable electronics [25]. Recently, organohydrogels exemplify a materials design paradigm where solvent‐mediated network engineering enables exceptional mechanical properties and environmental adaptability, oping new opportunities for reliable wearable electronics [18, 31, 32, 33]. Gu et al. developed a biocompatible organohydrogel based on an chitosan–lignosulfonate–gelatin network plasticized through ethylene glycol/water solvent, displaying good toughness (3.54 MJ/m^3^), exceptional fatigue resistance and subzero operation capability owing to its multiple interfacial interactions that promote efficient energy dissipation [18]. To achieve ultrastretchable property, Zhu et al. developed an organohydrogel by immersing a dual‑network PAM/PVA hydrogel matrix in a glycerol (Gly)/water binary solvent, exhibiting remarkable stretchability (5000 %), high optical transparency (78 %), and excellent freezing tolerance (–24°C), showing great potential for applications in self‐powered gesture recognition [31]. However, rational design of multifunctional electrode gel with finely tunable interactions and cross‐linking architectures to achieve soft yet tough mechanical properties is rarely reported.

In this work, we introduce a novel molecular chain lubrication strategy as a rational design to develop ultrasoft yet tough organohydrogels via one‐pot micellar polymerization method. Through Gly/water‐mediated dynamic lubrication for hydrophobically associated PAA/quaternized chitosan (QCS)/LiCl (HAPAA/QCS/LiCl) organohydrogel matrix (denoted as AQGL), the polymer‐polymer interactions and micellar cross‐linking structures within the gel were rationally modulated, achieving ultrasoft yet tough mechanical characteristics. Unlike conventional organic solvent‑soaking methods, which generally stiffen the gel network, our in situ lubrication strategy−employing a Gly/water co‑solvent during gelation and cross‑linking−constructs a gel network with dense reversible micelle cross‐linked architecture and abundant polymer‐solvent hydrogen bonding, achieving exceptional softness and toughness via enhanced energy dissipation. The as‐developed AQGL demonstrates ultrahigh stretchability (5800%), ultralow Young's modulus (14.3 kPa), remarkable toughness (11.77 MJ/m^3^), and high fracture energy (75.96 kJ/m^2^), which is closely mimicking the compliant and resilient nature of human skin. Beyond its mechanical robustness, the AQGL exhibits versatile functional characteristics, including high optical transparency (>90%), tunable ionic conductivity, impressive self‐adhesion, remarkable antibacterial activity, exceptional freezing tolerance (−40°C), notable anti‐icing capability, excellent water retention capacity (retaining ∼92% of its initial weight after 100 days), and good self‐healing property, revealing outstanding multifunctionality and environmental stability for wearable applications. To demonstrate the practical relevance, a flexible and durable single‑electrode TENG is constructed using AQGL as a transparent electrode together with an Ecoflex elastomer. The as‐fabricated AQGL‐TENG delivers a peak power density of 0.92 W/m^2^, showcasing great potential in wearable motion tracking, handwriting recognition, and autonomous energy harvesting. This study not only presents a new paradigm to break the trade‑off between stiffness and toughness via the molecular chain lubrication strategy, but also establishes a versatile platform of soft yet tough organohydrogels with multifunctional compatibility for next‐generation epidermal electronics.

## 2 Results and Discussion

### 2.1 Design and Characterizations of the AQGL Organohydrogel

**FIGURE 1:**
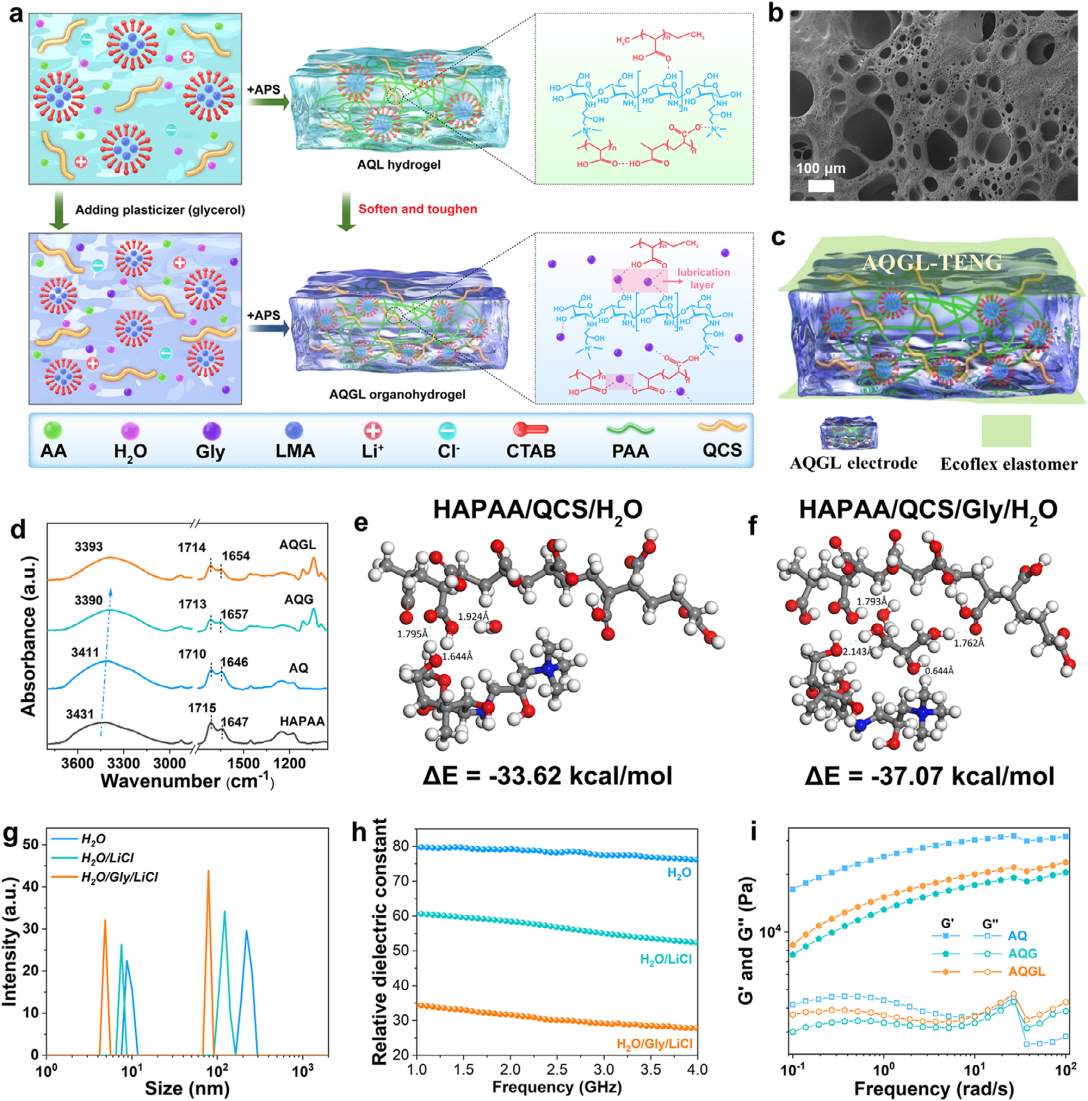
Manufacturing process and material characterization of the extremely ultrasoft yet tough AQGL organohydrogel for triboelectric applications. (a) Schematic diagram of the synthesis procedure, design strategy, and chemical structure of the AQGL organohydrogel. (b) SEM image of the AQGL organohydrogel. (c) Schematic diagram of the flexible AQGL‐TENG. (d) FTIR spectra of HAPAA, AQ, AQG, and AQGL gels. (e,f) DFT‐optimized structure and the interaction energy (ΔE) of HAPAA/QCS/H_2_O and HAPAA/QCS/Gly/H_2_O systems. (g) The size distribution of micelles in H_2_O, H_2_O/LiCl, and H_2_O/Gly/LiCl solutions. (h) Relative dielectric constant values of H_2_O, H_2_O/LiCl, and H_2_O/Gly/LiCl solutions. (i) Storage (G’) and loss modulus (G″) of AQ, AQG, and AQGL gels.

Figure 1a depicts the design strategy and synthesis procedures of the ultrasoft yet tough AQGL organohydrogels with multi‐functionality using a simple one‐pot micellar polymerization method. Specifically, the QCS, the hydrophilic monomer acrylic acid (AA), a small amount of hydrophobic monomer lauryl methacrylate (LMA), cationic surfactant cetyltrimethylammonium bromide (CTAB), and LiCl were initially dissolved in deionized water to form a homogeneous micellar solution. The water‐immiscible LMA, which possesses a lengthy hydrophobic alkyl side chain, was efficiently solubilized within the CTAB surfactant micelles. At this stage, the initiator ammonium persulfate (APS) can be incorporated into the micellar solution to initiate the micellar copolymerization to obtain the HAPAA/QCS/LiCl (AQL) hydrogel (Figure 1a, upper panel), in which the hydrophobic association micelles composed of hydrophobic domains and surfactants serve as the dynamic and reversible physical cross‐linking points. In this system, reversible micelle cross‐linking points will rapidly disassociate upon deformation to dissipate energies and gradually reconstruct after breaking, which can impart the hydrogel with mechanical toughness and self‐healing capability [17]. Nonetheless, strong polymer‐polymer interactions (hydrogen bonding and ionic interactions) between HAPAA and QCS chains inevitably stiffen the network in this aqueous system. To overcome the trade‐off between stiffness and toughness, Gly was introduced into the precursor solution as a plasticizer (Figure 1a, lower panel). The Gly/H_2_O clusters can efficiently insert into the polymer chains as the lubrication layer to minimize the strong polymer‐polymer interactions. Meanwhile, the hydroxyl groups (‐OH) of Gly promote abundant polymer–solvent interactions that can reversibly dissociate under deformation to dissipate energy [28]. Furthermore, with the presence of Gly, more hydrophobic micelles were formed within the precursor solution and the gel network, which in turn enhanced the energy dissipation capability of the obtained AQGL organohydrogel. Consequently, the incorporation of Gly as a plasticizer modulates the hydrogen‑bonding interactions and promotes the formation of a more dynamic cross‐linking structure, which facilitates efficient energy dissipation and thereby simultaneously softens and toughens the flexible transparent gel (vide infra).

As depicted in Figure 1b, SEM images demonstrate that the as‐prepared AQGL organohydrogel showcases porous yet interconnected morphology with hierarchical structure. Within the hierarchical structure, water channels enable efficient transport of Li^+^ and Cl^−^ ions, contributing to the high ionic conductivity of AQGL gels. Experimental measurements demonstrate that the conductivity can be tuned from 0.11 to 1.84 S/m as the LiCl concentration rises from from 0 to 1 m (Figure S1). Accordingly, the as‐developed AQGL organohydrogel can serve as the transparent electrode integration for wearable TENGs (Figure 1c). To gain deeper insight into the role of individual components in the hydrogel network, the intermolecular interactions were investigated by attenuated total reflection Fourier transform infrared (ATR‐FTIR) spectroscopy. In the HAPAA/QCS hydrogel (denoted as AQ hydrogel for brevity) with pure water as the solvent, strong hydrogen bonding interactions and ionic interactions were formed between the HAPAA and QCS polymer chains [34]. As shown in Figure 1d, the broad peak at 3431cm^−1^ in the HAPAA hydrogel (black curve) exhibits a significant redshift to 3411 cm^−1^ in the AQ hydrogel (blue curve), which is attributed to the overlapping of ‐OH stretching vibration peak of HAPAA and H_2_O. In addition, the C = O stretching vibration peak of HAPAA (1715 cm^−1^) also shifts to a lower wavenumber (1710 cm^−1^) in AQ hydrogel. These results confirm that the robust interactions between HAPAA and QCS polymer chains can strongly bridge them, severely restricting their mobility and consequently stiffening the gel while compromising its stretchability and toughness [35]. Herein, inspired by the “plasticizing effect” generally applied in plastic processing, the Gly has been added as the plasticizer into the precursor solution to break the limitations of mechanical properties in AQ hydrogel. After incorporating the Gly, the ‐OH bending vibration peak of H_2_O in AQ hydrogel at 1647 cm^−1^ reveals blueshifte to 1657 cm^−1^ (green curve), implying the formation of Gly/H_2_O clusters in the HAPAA/QCS/Gly organohydrogel (denoted as AQG). In addition, there is a redshift observed in the ‐OH overlapping stretching vibration peak from 3411 to 3390 cm^−1^, indicating the enhanced interactions within the AQG organohydrogel compared with the AQ hydrogel. However, it can be noted that a blueshift in C = O stretching vibration peak of HAPAA (from 1710 to 1713 cm^−1^) is also observed, suggesting reduced interaction strength between HAPAA chains and other components in the organohydrogel.

To further understand this phenomenon, the interactions within AQ and AQG gels were simulated through the DFT calculations. As shown in Figure 1e, the calculated DFT results display that the strong hydrogen bonding interactions existed between HAPAA and QCS chains in the AQ hydrogel, exhibiting a total interaction energy of −33.62 kcal/mol, which is consistent with the FTIR results. As for the AQG systems (Figure 1f), the optimized structure demonstrates that the Gly/H_2_O cluster with much bigger size than H_2_O molecules can efficiently insert into the polymer chains, which can act as plasticizer to enlarge the distance between adjacent polymer chains, minimizing the strong interactions among polymer chains and thus enhance their flexibility. Meanwhile, the Gly/H_2_O cluster can bridge polymer chains via multiple hydrogen bonding interactions. Accordingly, more hydrogen bonds can be formed between Gly and H_2_O, as well as between Gly/H_2_O and polymer chains within the AQG gel, endowing the AQG systems higher total interaction energy (−37.07 kcal/mol) than the AQ system, which leads to a redshift of the ‐OH stretching vibration peak to lower wavenumber (Figure 1d). Importantly, polymer‐solvent hydrogen bonding interactions between solvent molecules (e.g., Gly/H_2_O) and polymer chains do not markedly impede chain mobility or stiffen the gel matrix, as the high mobility of small solvent molecules and the facile slippage of polymer chains facilitate the dissociation of these interactions under deformation [36]. The disassociation of these hydrogen bonds also dissipates significant amount of deformation energies [16]. Thus, the hydrogel exhibited simultaneous softening and toughening effects with the incorporation of Gly as the solvent. Meanwhile, the introducing of LiCl also alters the interactions within the gel. With the presence of LiCl (orange curve), the ‐OH stretching vibration peak undergoes a blueshift toward higher wavenumber, while accompanied by a slightly redshifted ‐OH bending vibration peak (Figure 1d), suggesting the reduced interactions between ‐OH groups in the AQGL organohydrogel compared to the AQG gel. These phenomena can be attributed to the strong hydration capability of Li^+^, which disrupts the hydrogen bonding between Gly and H_2_O molecules, thereby further weakening the hydrogen bonds among the polymer chains [37].

In addition to altering the interactions within the AQ hydrogel, it can be found that the incorporation of Gly and LiCl also modulates the cross‐linking structure of the gel. As depicted in Figure 1g, dynamic light scattering (DLS) measurements indicate the presence of two distinct micelle populations with average hydrodynamic diameters of 220.2 and 8.7 nm in the precursor solution prepared with pure water as the solvent, which can be attributed to CTAB/LMA and pure CTAB micelles, respectively. The above values sharply drop to 122.4 and 7.5 nm upon LiCl addition, while further decline to 78.8 and 4.8 nm with subsequent Gly incorporation. The reduction in micelle size originates from the decreased dielectric constant of water induced by Gly and LiCl (Figure 1h). This reduced polarity enhances electrostatic interactions and suppresses the aggregation of ionic surfactants, leading to an increased critical micelle concentration (CMC). Additionally, the incorporation of Gly reduces solvent cohesiveness, enhancing the solubility of hydrocarbon tails in hydrophobic monomer and surfactant molecules, which diminishes solvophobic interactions and promotes the formation of smaller micelles with lower surfactant aggregation numbers [38, 39]. Nevertheless, it can be found that the peak intensity of the LMA/CTAB micelles is significantly heightened after the incorporation of LiCl and plasticizer Gly (Figure 1g), indicating that more hydrophobic micelles with smaller size are formed in the precursor solution of the AQGL organohydrogel, which enhances energy dissipation capability of the gel. Remarkably, the increased micellar cross‐linking points have a limited effect on the gel's modulus, since they readily dissociate under deformation even at small strains [12].

Rheological experiments were subsequently conducted to investigate the dynamic viscoelastic properties of these gels and to further validate the aforementioned analysis. As depicted in Figure 1i, both the storage (G') and loss (G'') modulus of AQG are significantly lower than those of AQ. As observed, Gly exerts a plasticizing effect by separating polymer chains, which lubricates the gel network and decreases the interchain frictions between adjacent polymer chains. Additionally, G′ and G″ values of AQGL are slightly higher than those of AQG, suggesting that LiCl incorporation mildly reinforces the gel. This effect is likely due to the formation of coordination lithium bond between Li^+^ ions and carbonyl groups on the polymer chains, which possess partial covalent character and are considerably stronger than hydrogen bonds, thus efficiently strengthening the gel matrix [40, 41]. Significantly, our molecular chain lubrication strategy is fundamentally differs from the previously reported solvent exchange methods to incorporate polyols (e.g., Gly, ethylene glycol) into the gel matrix. Generally, Gly introduced via solvent exchange typically fails to break the strong interactions that have already formed between polymer chains. Additionally, newly established hydrogen bonds between polymer chains and polyol/H_2_O clusters act as the additional cross‐linking sites, inducing gel shrinkage and reducing the polymer chain flexibility, which will finally stiffen the hydrogel network [17, 20]. As a result, significant matrix contraction is observed when the AQ hydrogel is immersed into Gly, leading to an enhanced rigidity and mechanical reinforcement of the gel (Figure S2).

### 2.2 Mechanical Properties

**FIGURE 2:**
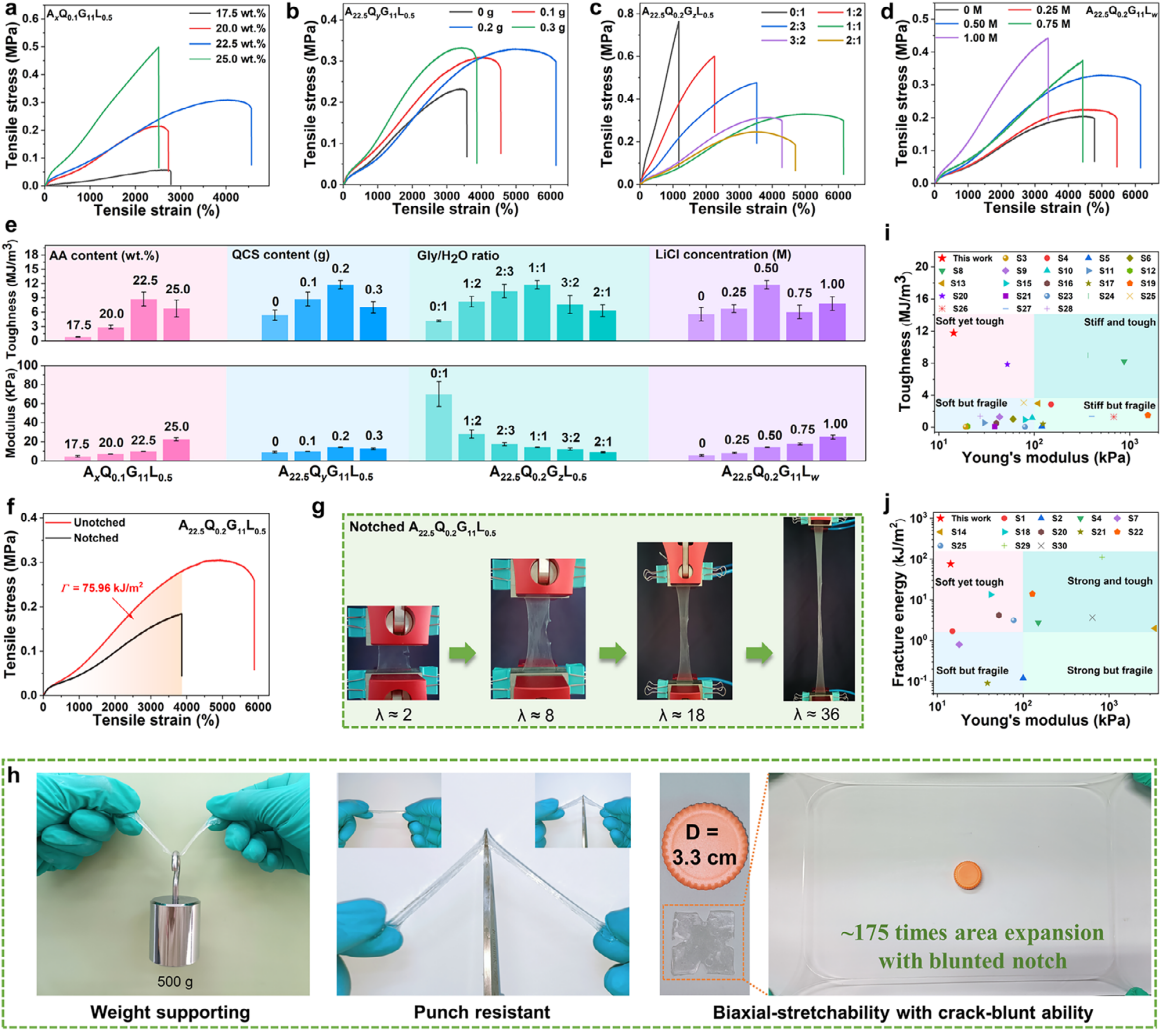
The mechanical properties of AQGL organohydrogels. (a) Representative tensile stress–strain curves of (a) A*_x_* Q_0.1_G_11_L_0.5_, (b) A_22.5_Q*_y_* G_11_L_0.5_, (c) A_22.5_Q_0.2_G*_z_* L_0.5_, and (d) A_22.5_Q_0.2_G_11_L*_w_* organohydrogels. (e) Summary of the toughness and Young's modulus of AQGL organohydrogels with different compositions. (f) Representative tensile stress–strain curves of the pre‐notched A_22.5_Q_0.2_G_11_L_0.5_ sample. (g) Photographs showing the excellent crack‐blunting capability of the A_22.5_Q_0.2_G_11_L_0.5_ organohydrogel. Comparing parameters of (i) toughness versus modulus and (j) fracture energy versus modulus between AQGL organohydrogel and other recently reported gels. (h) Photographs vividly show the outstanding mechanical properties including weight‐supporting ability, punch‐resistant, biaxial‐stretchability, and crack‐blunting capability of the AQGL organohydrogel.

As the mechanical properties of the AQGL organohydrogel are highly dependent on the composition, we optimized the mechanical performances of the AQGL organohydrogels by systematically tuning their compositions (Table S1). The obtained AQGL gel is denoted as A*_x_* Q*_y_* G*_z_* L*_w_* for brevity, where *x*, *y*, *z*, and *w* represent the AA content (wt.%), QCS weight (g), Gly/H_2_O weight ratio and LiCl molar concentration (M), respectively. Fine‐tuning the synthesis conditions enable the AQGL organohydrogel to exhibit highly tunable mechanical properties including tensile strength (0.061 ∼ 0.563 MPa), stretchability (1119% ∼ 5800%), Young's modulus (4.7 ∼ 70.0 kPa), toughness (0.85 ∼ 11.77 MJ/m^3^), compression strength (0.85 ∼ 3.02 MPa, at compression strain of 0.90) and compression modulus (14.3 ∼ 84.2 kPa) (Figure 2a–e; Figures S3–S5, and Tables S2–S5). As shown in Tables S2 and S3, both the tensile and compressive strength and modulus progressively increase with higher AA (x ≤ 22.5) and QCS (y ≤ 0.2) contents due to enhanced network density and stronger chain entanglement between HAPAA and QCS. Nevertheless, excessive AA (x > 22.5) and QCS (y > 0.2) contents result in reduced stretchability and toughness due to the restricted chain mobility within the over‐densified gel network. Additionally, owing to the lubrication effect of the plasticizer Gly molecules, the gel exhibits progressive softening accompanied by increased toughness as the Gly content increases (z ≤ 1:1), as evidenced by reduced strength and modulus alongside enhanced stretchability and toughness. Furthermore, increasing LiCl concentration progressively enhances the gel's strength and modulus due to the formation of strong lithium coordination bonds. Meanwhile, stretchability and toughness reach a maximum at a specific LiCl concentration of 0.5 m, while declining thereafter. At lower LiCl levels, additional hydrophobic micelle cross‐links contribute to energy dissipation. Yet at higher LiCl concentrations, enhanced energy dissipation becomes insufficient to offset the rigidity induced by excessive lithium bonding, ultimately compromising the molecular chain flexibility. As a result, the A_22.5_Q_0.2_G_11_L_0.5_ organohydrogel demonstrates the optimal mechanical properties, characterized by exceptional stretchability (5800%) and toughness (11.77 MJ/m^3^), and moderate tensile strength (0.331 MPa). Notably, the as‐developed organohydrogel simultaneously exhibited an ultra‐low Young's modulus (14.3 kPa), which is comparable to the level of human skin [42]. Thus, the A_22.5_Q_0.2_G_11_L_0.5_ organohydrogel has been selected for the following investigations unless otherwise stated.

The fracture energy of the AQGL organohydrogel was then measured using the well‐established single‐notch method (Videos S1 and S2). As displayed in Figure 2f,g, the pre‐made crack did not advance into the AQGL organohydrogel even at extremely high strain (λ ≈ 36), exhibiting a high fracture energy up to 75.96 kJ/m^2^ that is far beyond that of the biological tissues (≤30 kJ/m^2^), which is attributed to its strong crack‑blunting capability arising from efficient energy dissipation [15]. Meanwhile, the as‐measured value in this work is far beyond that of most reported tough gels (Table S6). These intriguing biomimetic soft yet tough mechanical features make the AQGL organohydrogel easily deform yet hard to broken. As vividly presented in Figure 2h, the A_22.5_Q_0.2_G_11_L_0.5_ organohydrogel is punch resistant and capable of supporting a 500 g weight (over 1000 times the gel's weight) without any damages. Additionally, a small notched rectangle sample can be easily biaxial‐stretched to ∼175 times its initial area without broken, further highlighting the excellent stretchability, crack‐blunting ability, and soft yet tough mechanical features.

Cyclic tensile loading‐unloading tests were conducted to evaluate the energy dissipation behavior of AQGL organohydrogels. The energy dissipation capacity per unit volume was quantified by the hysteresis area enclosed between the loading and unloading curves [15]. As shown in Figure S6a,b, The results demonstrate that incorporate an optimal amount of glycerol (z = 1:1) into the AQL gel matrix significantly enhances the energy dissipation capacity of the network, whereas excessive glycerol content (z = 2:1) results in a reduction of this property (Figure S6a,b). This behavior is attributed to a substantial decrease in the number of hydrophobic micelles in the precursor solution at higher glycerol concentrations (z = 2:1), which likely arises from the significantly increased critical micelle concentration (CMC) under high glycerol conditions (Figure S6c) [38]. Furthermore, the energy dissipation capacity of the AQGL organohydrogels was observed to increase with elevated LiCl concentrations, owing to the enhanced formation of hydrophobic micelles within the gel network (Figure S6d,e). These findings further verify the above discussions and confirm that the incorporation of both glycerol and LiCl into the AQ gel network improves its energy dissipation capability by promoting reversible hydrophobic micelle‐based cross‐linking, thereby enhancing the overall toughness and fracture energy of the gels.

To date, reconciling the conflict between stiffness and toughness remains a persistent challenge in developing artificial soft tissue–mimetic materials [21]. Nonetheless, we demonstrated here that the proposed molecular chain lubrication strategy could simultaneously enhance the softness and toughness of the organohydrogel. For comparison, the toughness and fracture energy against Young's modulus of the as‐developed AQGL gel and recently reported tough gel materials are plotted in Figure 2i,j, and the corresponding summarized data can be found in Table S6. Apparently, the as‐proposed organohydrogel with a considerably high toughness and fracture energy ranks into the top‐class tough gels while maintaining an extremely low modulus. These exceptional mechanical characteristics resemble biological soft tissues make the AQGL organohydrogel an ideal matrix for soft electronics. For instance, the low modulus can minimize mechanical mismatch between electronics and soft tissues, facilitating accurate signal acquisition and transmission. Meanwhile, high toughness and fracture energy can efficiently prevent the dysfunction of electronics from mechanical damage, thus maximizing the service life of the gel electronics, prolonging the operational lifespan of gel‐based devices.

### 2.3 Multifunctional Characteristics of the AQGL Organohydrogel

**FIGURE 3:**
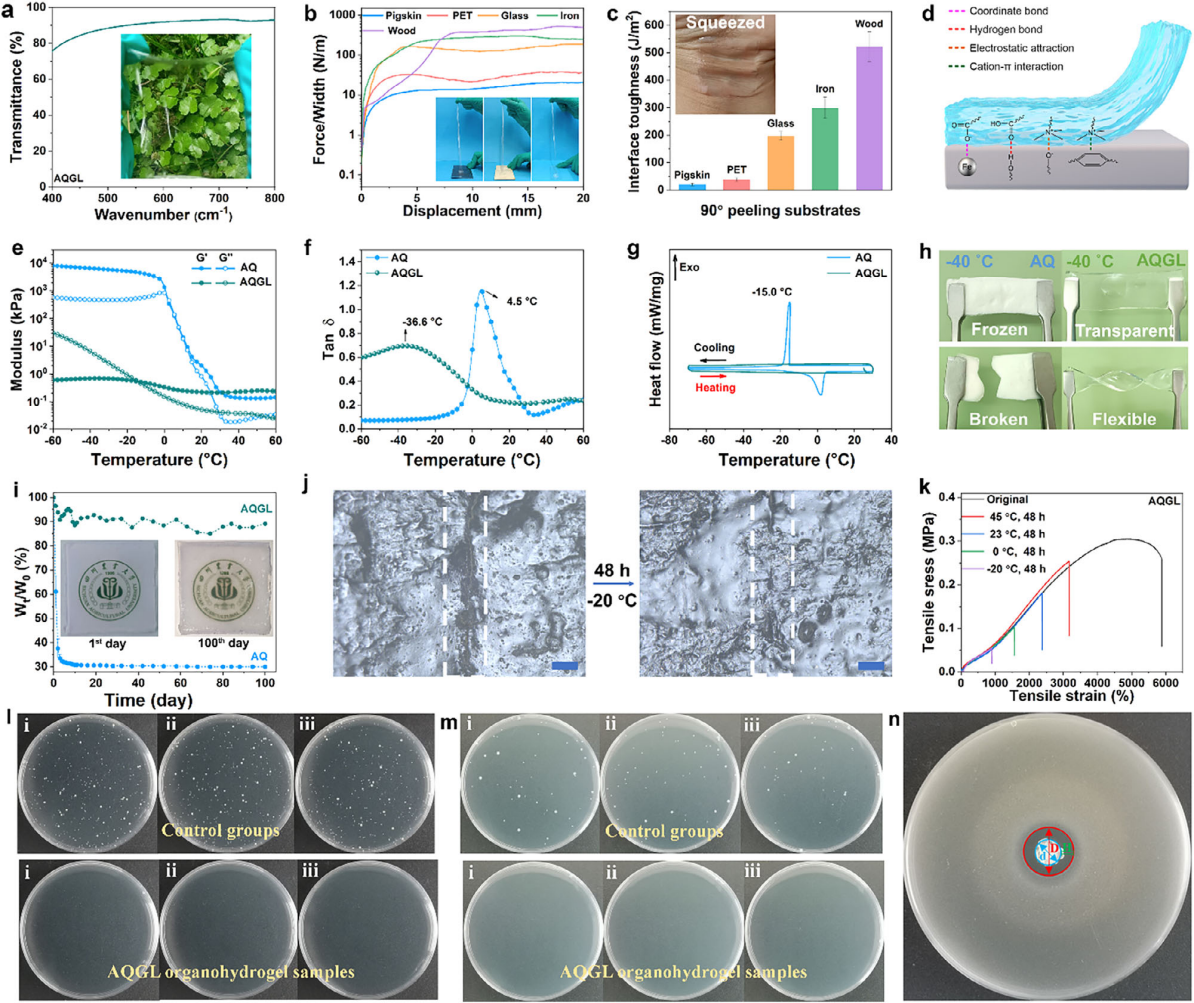
Multi‐functionality of the AQGL organohydrogel. (a) Optical transmittance spectrum of AQGL hydrogel (∼2 mm thick) in the visible range. (b) Force‐displacement curves via representative 90°‐peeling test. (c) Calculated interface toughness of the AQGL organohydrogel on diverse substrates. (d) Adhesion mechanism of the AQGL organohydrogel. (e, f) DMA and tan δ curves of AQ and AQGL gels in the temperature range of −60°C–60°C. (g) DSC curves of AQ and AQGL gels within the temperature range of −70°C–30°C. (h) photographs of AQ and AQGL samples at −40°C. (i) Mass retention profiles of AQ and AQGL hydrogels under ambient conditions over 100 days. (j) Laser confocal microscopy images of the notched and healed AQGL organohydrogel (Scale bar: 50 µm). (k) Representative tensile stress–strain curves of the AQGL organohydrogel before and after self‐healing at different temperatures for 48 h. (l,m) Comparative antibacterial performance against *S. aureus* (l) and *E. coli* (m) for AQGL and control samples. (n) Corresponding inhibition zone against *S. aureus* (sample diameter d: 5 mm).

In addition to its soft yet tough mechanical properties, the AQGL organohydrogel also exhibits remarkable multifunctionality. The incorporation of Gly and LiCl significantly diminishes the size of hydrophobic association micelles and facilitates the uniform hybridization of HAPAA and QCS chains by spatially separating them, which will effectively reduce the light scattering. Consequently, the AQGL organohydrogel displays a much higher transparency than AQ and AQL hydrogels (Figure S7). Namely, the transparent AQGL gel plate appears ∼95% transmittance in the visible range even at a thick thickness of 2 mm (Figure 3a). Self‐adhesion capability is another crucial functionality for gel materials, enabling the construction of intimate HMIs and stable interfacial contact in TENG devices. As measured by the 90° peeling tests, the interfacial adhesion toughness of AQGL organohydrogel on glass and wood reaches up to 198 and 521 J/m^2^, respectively (Figure 3b,c). Particularly noteworthy that the AQGL gel can self‐adhere on human joint and conformally deform with human skin and joint (Figure 3c; Figure S8), highlighting its significant application potential in epidermal soft electronics. The AQGL organohydrogel contains abundant functional groups including polar ‐COOHs and ionic ‐COO^−^, which can facilitate strong interfacial interactions (e.g., hydrogen bonding and metal coordination) with various substrates (Figure 3d). In addition, the positively charged quaternary ammonium groups of QCS chains capable of attaching to the materials surface via electrostatic attractions and cation‐*π* interactions. Furthermore, the extreme softness of the gel may facilitate the penetration of polymer chains into the micropores of rough surfaces, forming mechanically interlocked structures. Benefiting from its integrated properties, the AQGL organohydrogel exhibits strong adhesion to a broad range of substrates, including plastics, rubbers, metals, glasses, and biological surfaces such as human skin and wet tissues (kidney and heart) (Figure S9).

The incorporation of Gly and LiCl also synergistically endows the AQGL organohydrogel with desired freezing and drying tolerance, which is crucial for its long‐term utilization in cold conditions. In detail, the hydrogen bonding between Gly and H_2_O molecules would compete with that between H_2_O molecules, thus disrupt the stable structure of H_2_O molecules and impedes water crystallization at low temperatures [17, 33]. As for the role of LiCl, on the one hand, the incorporation of LiCl can significantly increases the concentration of solute particles, causing the decrease of freezing temperature of solute; on the other hand, the hydration effect between ions and H_2_O molecules would reduce the free H_2_O molecules concentration in the liquid phase and thus reduce the ice‐water equilibrium point to lower temperatures [43]. Furthermore, the abundant Li^+^ and Cl^−^ ions within the solute can remarkably decrease its saturated vapor pressure, thus inhibiting water evaporation. As such, the incorporated glycerol molecules and LiCl can efficiently suppressing both water crystallization in cold environment and evaporation under room temperature conditions. Herein, the dynamic mechanical analysis (DMA) (Figure 3e,f) and differential scanning calorimetry (DSC) (Figure 3g) methods were combined to assess the anti‐freezing capability of the AQ (A_22.5_Q_0.2_) and AQGL gels. As anticipated, the AQ hydrogel readily freezes at −15.0°C and exhibits a more than 10^5^ times increase in the storage modulus (*G’*) upon cooling to −60°C due to the absence of anti‐freezing mechanisms. In stark contrast, no exothermic peak was observed in the DSC cooling curve of the AQGL organohydrogel within the test temperature range from −70°C to 30°C, and only a threefold enhancement in G’ values were observed during the same cooling process. Furthermore, as shown in the tan δ curves (Figure 3f), the phase transition temperature of the AQ hydrogel is observed at approximately 4.5°C, whereas that of the AQGL organohydrogel is significantly reduced to around −36.6°C. Consequently, the AQGL organohydrogel maintains its stretchable and twistable mechanical properties even at extremely low temperature of −40°C (Figure 3h; Figure S10) and maintains its high transparency even at −60°C (Figure S11), while the AQ hydrogel has been already frozen into an opaque and fragile solid at −40°C (Figure 3h). Besides, the anti‐icing performance of AQGL was evaluated using a thermoelectric cooler at a constant temperature of −15°C. As shown in Figure S12, real‐time monitoring results reveal that the −15°C aluminum plate can induce ice formation from ambient water vapor within 20 min, while the anti‐freezing AQGL organohydrogel can effectively guarantee the material surface and the covered region to remain ice‐free. After continuous testing for 1 h, the AQGL maintains excellent flexibility and optical transparency, highlighting its strong potential for wearable electronics in cold environments. Meanwhile, the AQGL organohydrogel exhibits excellent drying resistance, retaining ∼92% of its initial weight and maintaining its physicochemical properties (e.g., electrical conductivity and mechanical integrity) after 100 days of ambient storage (∼65% RH, 25°C) (Figure 3i; Figures S13 and S14). For instance, the AQGL organohydrogel still enjoys high tensile strain of 4943 ± 48% and maintains 89.4% of its initial toughness after 100 days (10.25 ± 0.12 MJ/m^3^). In contrast, the AQ hydrogel lost approximately 70% of its weight within 20 days, accompanied by a substantial reduction in electrical conductivity (Figure S10).

Benefiting from the dynamic cross‐linking structures, the AQGL organohydrogel also exhibits an autonomous self‐healing capability. Upon mechanical damage, the hydrophobic association micelles and polymer–solvent interactions at the fractured interfaces inevitably dissociate. When the separated pieces are reassembled, the dissociated CTAB molecules gradually migrate toward the interface, where the hydrophobic segments of HAPAA chains re‐engage with these surfactants, leading to the regeneration of hydrophobic micelles that act as new cross‐linking nodes [44]. In addition, the hydrogen bonding between polymers and Gly/H_2_O also gradually reassociates. As such, the AQGL organohydrogel was automatically self‐healed. Importantly, the self‐healing capability of the gel can be retained in cold environments, since the anti‐freezing capability can guarantee the mobility of polymer chains and surfactants at low temperatures. Intuitionally, the damage interface grew together after healing at −20°C for 48 h (Figure 3j), and the healed sample can be stretched to ∼980% strain (Figure 3k). Interestingly, this value was gradually enhanced with increasing the healing temperature as the accelerated mobility of surfactants and polymer chains at high temperatures. For instance, after healing at approximately 23°C for 48 h, the recovered sample achieves a stretchability of around 2500%, which is sufficient to ensure reliable performance in diverse practical scenarios. To further demonstrate the self‐healing capability of the AQGL organohydrogel, gel samples were dyed in various colors. Using these colorful gels, complex shapes and structures can be readily assembled, such as a mushroom, a peach, and the surface of a Rubik's cube (Figure S15). Owing to the good self‐healing capability, the AQGL gel displays the ability to support electrical current after damage (Figure S16).

Moreover, antibacterial evaluations of the AQGL organohydrogel against *Staphylococcus aureus (S. aureus)* ATCC 6538 and *Escherichia coli* (*E. coli*) ATCC 8739 were conducted to assess its wearable safety for applications in wearable electronic skin. As shown in Figure 3l, the colony counts of *S. aureus* in the three control groups are 371, 366 and 362 CFU, while those on AQGL samples are all below the detectable limit (<1 CFU). Similarly, for *E. coli*, the control groups exhibit high colony count of 51, 56 and 53 CFU, whereas the treated samples consistently show values below 1 CFU (Figure 3m). According to the calculation formulas (details can be found in the Experimental Section) [45], the antibacterial rate and antibacterial activity of the AQGL sample against *S. aureus* are 99.9999% and 7.57, while those against *E. coli* are 99.9999% and 6.72. In addition, the inhibition zone diameter (D) of the AQGL gel sample against *S. aureus* is 14.4 mm (Figure 3n), while that against *E. coli* is 8.3 mm (Figure S17). The antibacterial test results against *S. aureus* and *E. coli* have been summarized in Table S7, indicating distinguished antibacterial properties of the as‐developed gel sample. In addition, the AQGL also exhibits good in vitro cytocompatibility as the MC3TC‐L1 cells cultured on gel surface for 1, 4, and 7 days both displayed almost entirely green florescence and exhibited high cell viability of 96.73%, 92.11, and 87.64%, respectively (Figure S18).

### 2.4 Triboelectric Properties of the AQGL‐TENG

**FIGURE 4:**
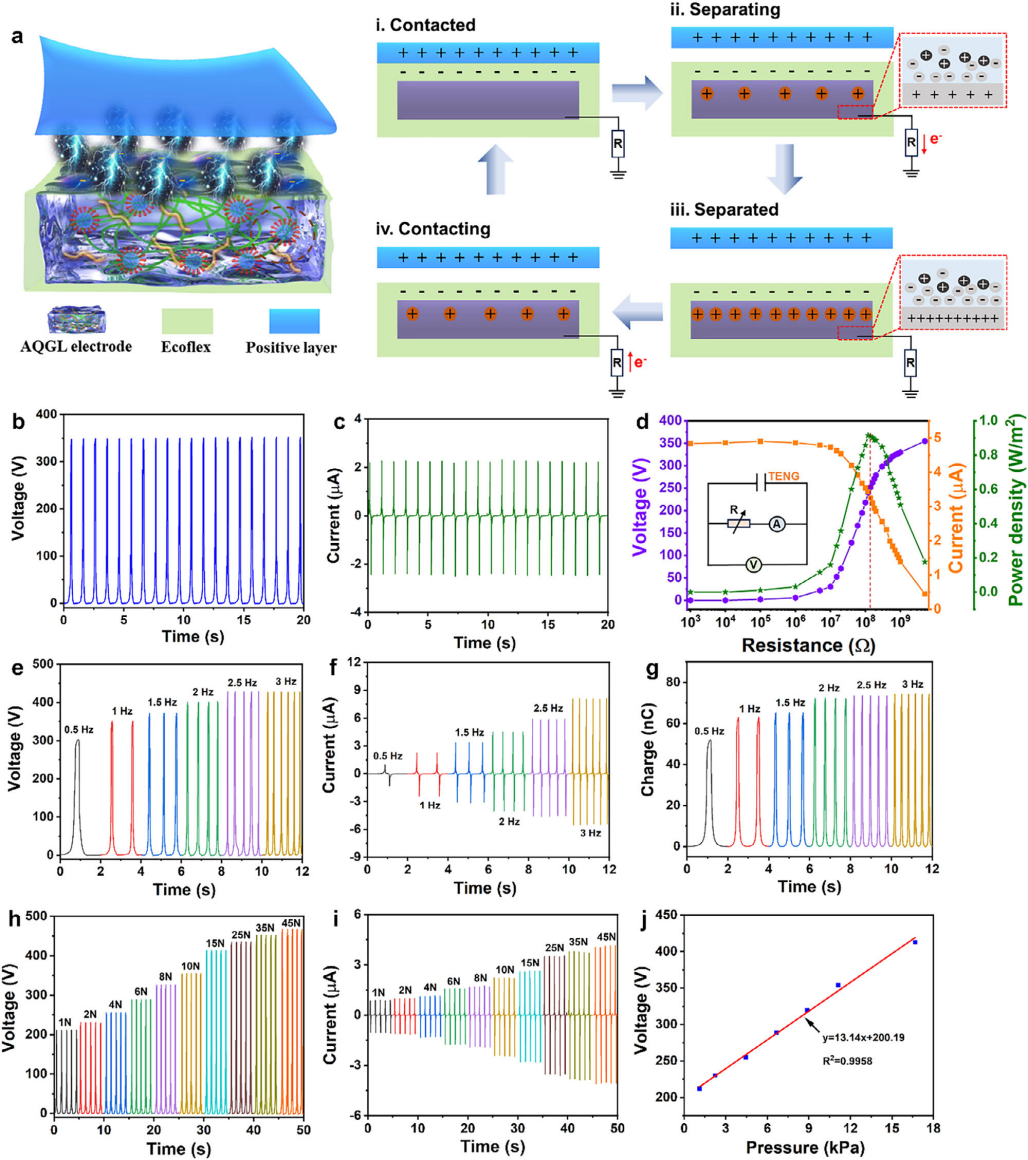
Working mechanism and electrical characterization of the AQGL‐TENG. (a) Schematic depiction of the single‐electrode mode AQGL‐TENG operation. (b,c) Generated open‐circuit voltage and short‐circuit current under constant mechanical loading (10 N, 1 Hz). (d) Influence of external load resistance on output voltage, current, and power density. (e–g) Response of open‐circuit voltage, short‐circuit current, and transferred charge to varying excitation frequencies. (h,i) Output voltage and current at different mechanical loading forces. (j) Linear fitting relationship between generated voltage and applied pressure (1–17 kPa).

Owing to its multifunctional features including good conductivity, self‐healing, freezing/drying tolerance, optical transparency, adhesiveness, and antibacterial activity, as well as the soft yet tough mechanical features, the AQGL organohydrogel holds great promise for enhancing the performance of gel‐based TENGs and advancing their applications in energy harvesting and wearable electronics. To investigate the applicability of the AQGL‐based TENG (AQGL‐TENG) in epidermal electronic systems, a flexible device (3 cm × 3 cm) in a single‐electrode model was fabricated by integrating a transparent AQGL hydrogel electrode with the Ecoflex elastomer (Figure 4a). Upon contact with an external object that has a stronger tendency to donate electrons than Ecoflex, electron transfer will occur across the interface, resulting in Ecoflex gaining electrons and becoming negatively charged. Here, a polyethylene (PE) film is applied as another triboelectric layer to evaluate the energy harvesting capability of the AQGL‐TENG.

During contact, the PE film and Ecoflex elastomer acquire equal but opposite triboelectric charges on their surfaces (Figure 4a, i). As they separate, the electrostatic field from the charges on the insulating Ecoflex induces the migration of mobile ions within the AQGL hydrogel. This forms an ionic shielding layer at the hydrogel‐elastomer interface that electrostatically screens the bound surface charges (Figure 4a, ii). Concurrently, an electrical double layer (EDL) is established at the metal–electrolyte interface, with an excess of negative ions in the hydrogel inducing positive charges in the metal electrode [46]. To neutralize this induced potential, electrons flow from the electrode to the ground through the external circuit until electrostatic equilibrium is reached (Figure 4a, iii). When the PE and Ecoflex are brought back into contact, the process reverses, driving electrons back through the external load (Figure 4a, iv). This periodic contact–separation cycle, combining contact electrification and electrostatic induction, thereby generates a sustainable alternating current. Moreover, mechanical deformations caused by human motion or environmental vibrations further induce repeated contact and separation between Ecoflex and other object surface such as human skin, which can convert wearable mechanical stimuli into electrical outputs.

The as‐developed AQGL‐TENG delivers remarkable electrical outputs, including an open‐circuit voltage of 352 V, a short‐circuit current of 4.5 µA and a transferred charge of 62.8 nC under a fixed mechanical input of 10 N at 1 Hz (Figure 4b,c; Figure S19). Besides, the AQ‐TENG presents an open‐circuit voltage of 304 V and a short‐circuit current of 2.6 µA (Figure S20). Though gel network optimization and ionic conductivity improvement of the AQGL, the output voltage and current of the AQG‐TENG increased by 15.79% and 73.08% respectively compared to those of the AQ‐TENG. These outputs are sufficient to instantaneously power an “IOE” pattern composed of 80 LEDs (Video S3 and Figure S21), demonstrating efficient energy transduction. The stability and uniformity of the generated output signals further highlight the AQGL‐TENG's high reliability and repeatability. Moreover, the AQGL‐TENG achieves a peak power density of 0.92 W/m^2^ at an external load of 120 MΩ (Figure 4d), highlighting its strong energy‐harvesting capability. To assess the energy harvesting performance under varying frequencies, outputs were measured across a range of 0.5–3 Hz (Figure 4e–g; Figure S22). The output voltage and transferred charge show minimal variation with increasing frequency, while the output current increases markedly from 2.29 to 13.51 µA due to reduced charge transfer duration at higher frequencies, which is consistent with the phenomenon in recently reported references [47, 48, 49]. These results emphasize the device's adaptability to irregular, low‐frequency motion typically encountered in wearable and ambient energy‐harvesting scenarios. To evaluate the effect of freezing temperature on the electrical output of the AQGL‐TENG, the device was subjected to freezing through a thermoelectric cooler at a constant temperature of −15°C for 1 h. As depicted in Figure S23, only the Ecoflex film will induce ice formation from ambient water vapor, but it can quickly recover flexibility after ice thawing. The whole AQGL‐TENG maintains good flexibility and a stable output voltage of 340 V (the surface temperature of the tested device is below 0°C after thawing), retaining more than 90% of its pre‐freezing performance, demonstrating great application potential of the AQGL‐TENG in low temperature conditions.

**FIGURE 5:**
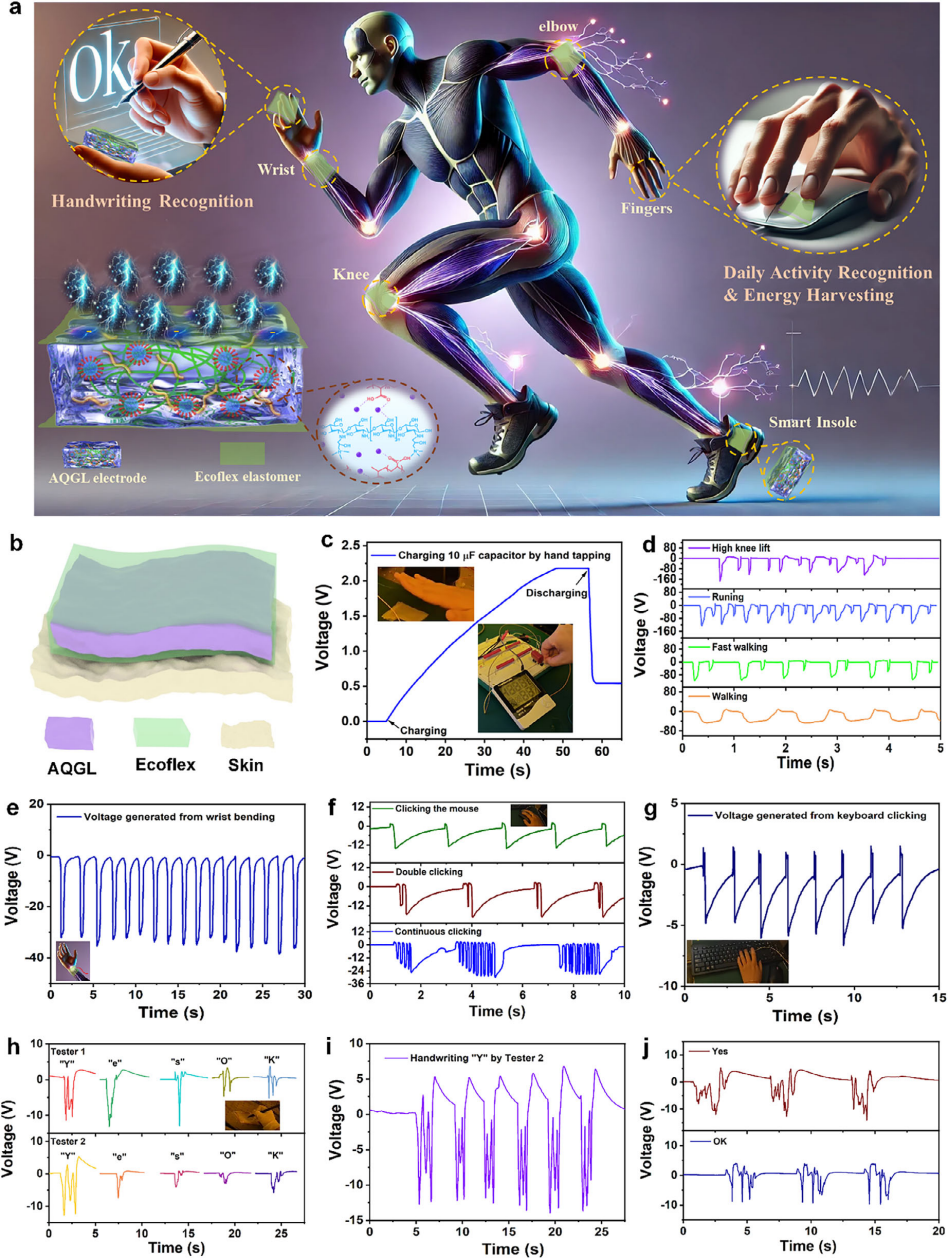
(a) Schematic representation of diverse wearable application scenarios. (b) Illustration of AQGL‐TENG attached to human skin. (c) Charging and discharging curves of a 10 µF capacitor powered by hand‐tapped AQGL‐TENG for driving a commercial thermo‐hygrometer. (d) Electrical output signals produced by the insole‐integrated AQGL‐TENG during various human activities. (e) Wearable energy harvesting from daily wrist bending. (f) Recognition of mouse‐clicking behavior. (g) Recognition of keyboard‐clicking behavior. (h‐j) Handwriting recognition tests of different letters written by various subjects.

The performance of AQGL‐TENG under various mechanical forces (1–45 N) at a fixed frequency of 1 Hz was also investigated (Figure 4h–j; Figures S24 and S25). A monotonic increase in output voltage (212–467 V), current (1.93–8.16 µA), and charge transfer (36.8–82.5 nC) has been observed, affirming its force‐responsive behavior. Besides, the voltage–pressure curve exhibits excellent linearity (R^2^  =  0.9958), and the sensitivity reached 13.14 V/kPa over a pressure range of 1–17 kPa (Figure 5i), indicating high potential for tactile sensing and pressure monitoring. Beyond energy generation, the AQGL‐TENG shows strong durability and functional stability for long‐term use. Over 14 000 continuous operation cycles at a constant driving frequency of 2 Hz, the device retained a consistent voltage output with negligible degradation (Figure S26), verifying its mechanical robustness and reliability. According to the long‐term stability test, the AQGL‐TENG maintains an output voltage of approximately 367 V after 100 days of storage (Figure S27), indicating a minimal fluctuation of only 4.26% compared to the original value, which can be attributed to the encapsulation function of the Ecoflex layer for the AQGL electrode that can effectively shields the device from environmental variations and preserves its performance over extended periods. To further evaluate the application stability, the AQGL‐TENG was tested under varying ambient temperature conditions. For temperatures ranging from 25°C to 65°C, the environment was controlled using a heating lamp, and the output voltage was measured at each stabilized point. Separately, for a high‐temperature endurance assessment, the device was subjected to 80°C for 30 min on a hot plate. As shown in Figure S28, the output voltage remained highly consistent throughout all tests, with a maximum fluctuation of only ≈4.36% even after the 80°C treatment. This temperature stability can be attributed to the low thermal conductivity of the Ecoflex film that can effectively block heat penetration, which will mitigate the direct thermal impact on the AQGL electrode. Additionally, test results demonstrate that the capacitors with varying capacitances (2.2, 4.7, 10, and 22 µF) can be charged to 15.44, 8.07, 4.51 and 2.86 V respectively by the AQGL‐TENG within 300 s driven by a constant mechanical force of 10 N at 1 Hz (Figure S29). Compared with other recently reported gel‐based TENGs, the proposed AQGL‐TENG demonstrates superior comprehensive performance in terms of mechanical properties, electrical conductivity, and output capability (Table S8), highlighting its promising potential as an efficient and wearable power source for next‐generation self‐powered systems. Based on the output performance comparison presented in Table S9, the AQGL‐TENG developed in this work demonstrates superior electrical outputs with multiple functionalities (e.g., self‐healing, anti‐freezing, transparency, and adhesion), demonstrating great application in wearable energy harvesting and motion monitoring.

### 2.5 Wearable Applications of the AQGL‐TENG

The practical applicability of the flexible AQGL‐TENG was evaluated for energy harvesting and motion sensing during repeated contact–separation interactions with human skin under realistic conditions. As illustrated in Figure 5a, the AQGL‐TENG enables a broad range of self‐powered applications relevant to daily human activities. The device adopts a multilayer structure by embedding the AQGL hydrogel electrode between two Ecoflex elastomer layers, forming a soft and skin‐conformal device with excellent stretchability and biocompatibility (Figure 5b). The incorporation of AQGL not only enhances the device's durability and mechanical compliance but also ensures long‐term wearing comfort and performance stability under dynamic deformation. The AQGL‐TENG (4 cm × 4 cm) was driven by hand tapping to evaluate its ability to harvest mechanical energy from human body motion. As shown in Figure 5c and Video S4, a 10 µF capacitor was charged to 2.2 V within 43 s, which is sufficient to power a commercial thermohygrometer. Furthermore, the as‐fabricated AQGL‐TENG can also transfer bending and stretching mechanical energy to electrical outputs. As demonstrated in Figure S30, the peak‐to‐peak output voltage of the AQGL‐TENG during one‐hand‐driven bending‐releasing cycles is about 120 V, while that of the AQGL‐TENG can reach up to almost 393 V when driven by two‐hand bending with higher mechanical force. Besides, the AQGL‐TENG device also generates a peak‐to‐peak voltage of 70 V under stretching‐releasing cycles, indicating the application potential for stretchable strain sensors. This result confirms its potential for harvesting mechanical energy to power small electronic devices without an external power source. In addition to energy harvesting, the AQGL‐TENG demonstrates sensitive motion detection when integrated with different body parts such as the insole, wrist, computer mouse, and keyboard. Distinct electrical signals were recorded during various gait modes, including walking, fast walking, running, and high knee lifting, which can clearly reflect movement intensity and pattern (Figure 5d; Video S5). Furthermore, signals captured during wrist flexion (Figure 5e), mouse‐clicking (Figure 5f), and typing on a keyboard (Figure 5g; Video S6) illustrate the device's utility in detecting subtle and repetitive activities, highlighting its capability for gesture monitoring and potential application in behavioral analytics, human‐machine interaction, and health monitoring.

The multifunctionality of AQGL‐TENG can be further exemplified as a flexible electronic screen for real‐time handwriting recognition, which demonstrates an exceptional responsiveness in tracking different handwritten characters, enabling seamless interaction and feedback during the writing process (Figure 5h–j; Video S7). The capability of the AQGL‐TENG to detect and interpret dynamic, human‐generated motion in real time not only showcases its precision in motion sensing but also underscores its potential as a versatile interface for a wide range of interactive applications. The as‐presented functionality of the AQGL‐TENG opens exciting possibilities for next‐generation wearable technologies, where it can serve as a core component for interactive and self‐powered systems. The integration of handwriting recognition further enhances the potential of the AQGL‑TENG for realizing more intuitive, efficient, and user‑friendly systems in everyday applications. The developed AQGL‑TENG is expected to integrate energy‑harvesting, motion‑sensing, and interactive functions into a single flexible, self‑powered platform, which will facilitate the development of fully integrated wearable electronics.

## 3 Conclusion

In summary, we introduce a molecular chain lubrication strategy to overcome the long‐standing trade‐off between stiffness and toughness in multifunctional electrode gel. By incorporating Gly as a plasticizer, the intermolecular interactions between HAPAA and QCS polymer chains and the cross‐linking structure of the HAPAA matrix were effectively modulated, enabling the resulting AQGL organohydrogel exhibits an unusual combination of extreme softness and exceptional mechanical robustness. Beyond its mechanical superiority, the AQGL organohydrogel demonstrates a suite of integrated functionalities, including ionic conductivity, high optical transparency, self‐adhesive behavior, autonomous self‐healing, and remarkable resilience under subzero and arid conditions. It also exhibits inherent antibacterial properties, broadening its potential for biomedical and wearable applications. Moreover, a flexible triboelectric nanogenerator (TENG) fabricated using the AQGL hydrogel delivers excellent electrical performance, supporting its applicability in real‐time biomechanical sensing, motion tracking, handwriting recognition, and sustainable energy harvesting. These findings present a promising direction for the development of next‐generation multifunctional hydrogels for self‐powered, flexible electronics.

## Conflicts of Interest

The authors declare no conflict of interest.

## Supporting information




**Supporting File**: advs74186‐sup‐0001‐SuppMat.docx.

**Supplementary Video 1**: advs74186‐sup‐0002‐VideoS1.mp4.

**Supplementary Video 2**: advs74186‐sup‐0003‐VideoS2.mp4.

**Supplementary Video 3**: advs74186‐sup‐0004‐VideoS3.mp4.

**Supplementary Video 4**: advs74186‐sup‐0005‐VideoS4.mp4.

**Supplementary Video 5**: advs74186‐sup‐0006‐VideoS5.mp4.

**Supplementary Video 6**: advs74186‐sup‐0007‐VideoS6.mp4.

**Supplementary Video 7**: advs74186‐sup‐0008‐VideoS7.mp4.

## Data Availability

The data that support the findings of this study are available from the corresponding author upon reasonable request.
