# Supplementary material for: Ultrasoft Yet Tough Multifunctional Organohydrogels Enabled by Molecular Chain Lubrication Strategy for Self‐Powered Wearable Electronics
Source: Adv Sci (Weinh). 2026 Jan 30;13(20):e22686. doi: 10.1002/advs.202522686 (PMC13067859; doi:10.1002/advs.202522686)
Supplement: Supplementary file 1 — Supporting File: advs74186‐sup‐0001‐SuppMat.docx. [file ADVS-13-e22686-s006.docx]

Supporting Information

**Ultrasoft yet Tough Multifunctional Organohydrogels Enabled by Molecular Chain Lubrication Strategy for Self-Powered Wearable Electronics**

*Si Wang, Lin Hu, Mingbo Pu, Mingfeng Xu,* *Tao Zhou, Hanbing Rao, Xiong Li, Xiaoliang Ma, Gehong Su*, Xiangang Luo**

**S1. Experimental Section**

*Materials:* AA, CTAB, LMA, and APS were obtained from Adamas-beta®. QCS was purchased from Macklin, while Gly and LiCl were supplied by Chengdu Kelong Chemical Reagent Co., Ltd. All reagents were used without further purification.

*Synthesis of the AQGL organohydrogel:* The QCS, AA, LMA, CTAB, and LiCl were initially dissolved in deionized water at 40 ℃ for 4 h to obtain a homogenous micellar solution. Subsequently, the Gly was added into the solution as plasticizer and stirred for another 2 h at room temperature to obtain the final precursor solution. Afterward, the initiator APS (0.5 wt.% relative to the total mass of AA and LMA) was added to the solution and stirred for 5 mins. Finally, the precursor solution was poured into a home-made PTFE mold and placed at 60 °C for 6 hours for polymerization. After polymerization, the gel plate can be peeled off from the mold.

*Characterization:* ATR-FTIR spectra were collected using a Nicolet iS50 spectrometer equipped with a ZnSe ATR crystal. UV–vis transmission spectra were obtained using a Lambda 950 spectrophotometer. Microstructural features were examined via SEM (JEOL JSM-5900LV). The sample for SEM was immersed in deionized water for 1 h before freeze-drying and then sputtered with gold before taking SEM images. DSC experiments of the gels were conducted from −70 to 30 °C at a heating/cooling rate of 5 °C min^-1^ under nitrogen (20 ml/min) on a DSC 204 (NETZSCH) differential scanning calorimeter. DMA measurements were conducted on a Q800 dynamic mechanical analyzer (USA) in tensile mode, applying a frequency of 1 Hz and a strain amplitude of 0.1% over a temperature range from −60 °C to 60 °C, with a controlled heating rate of 5 °C/min. The rheological behavior of the hydrogels was characterized using an AR 2000EX rheometer (TA Instruments) equipped with a parallel plate and Peltier system at room temperature, under a fixed strain of 1% across a frequency range of 0.1–100 rad/s. Laser confocal microscopy (LCSM, LSM700, Carl Zeiss, Germany) was employed to visualize the microstructure of samples post-cutting and healing, using a 405 nm laser (intensity: 5, pinhole: 1 Å). The relative dielectric constants were determined with a R60 vector network analyzer (Copper Mountain Technologies, USA) over the 1–4 GHz frequency range. Mechanical properties were tested on an Instron 5567 mechanical tester under both tensile and compressive modes. Swelling behavior, anti-freezing performances, long-term stability, self-healing performance, adhesion and antibacterial tests were evaluated under controlled conditions as described in detail in the Supporting Information. We confirm that informed written consent from all participants has been obtained.

*Swelling behavior*: The swelling ratio of the gels was measured by immersing the totally dried gel sample in deionized water at RT. The sample was removed from the water at regular intervals, and then recorded the gel’s weight after wiping off the water on the gel surface. The swelling ratio was calculated as (W_t_-W_0_)/W_0_, where W_0_ and W_t_ are the mass of the dry gel and at time t, respectively.

*Ionic conductivity*: The ionic conductivity of the ionogels was measured by the electrochemical impedance spectroscopy using an electrochemical workstation (CHI760E, CH instruments, China) operated in the frequency range of 0.01 Hz to 100 kHz with the amplitude of 5 mV. The ionic conductivity (σ) of the gel can be calculated *via* the following equation: σ = L/RS, where R, L, and S are the resistance obtained by the intercept at the real part in Nyquist plots, the length between two electrodes, and the contact area of the gel with electrodes, respectively. The electrical resistance change of the gel during deformation was measured in real-time using a two-point probe resistance measurement system of a Keithley 2601B source meter (USA).

*Mechanical Property*: All the mechanical tests (tensile and compression) were conducted on a versatile testing machine (Instron 5567, USA) with a 1 KN load cell. The tensile tests were performed with the dumbbell-like samples (4 mm in width, 50 mm in length, and 2 mm in thickness) with a fixed stretching speed of 100 mm min^-1^. The compression tests were carried out with cylinder samples at a constant compressing rate of 3 mm/min. The tensile strain (ε) was defined as the ratio of the gauge length (*L*) to the initial gauge length (*L_0_*, 15 mm) (*ε=(L-L_0_)/L_0_*), and the tensile stress (*σ*) was calculated by dividing the force (*F*) by the initial cross-section area (*A_0_*) of the sample (*σ=F/A_0_*). The elastic modulus (*E*) was calculated from the slope of the initial linear region of the stress-strain curve. The toughness (*T*) was calculated from the integrated area below the stress-strain curve up to the fracture point based on the following equation:

$$T= \int_{\varepsilon_{0}}^{\varepsilon_{f}} \sigma\left( \varepsilon\right)d\varepsilon$$

where *ε_0_* and *ε_f_* represent the initial strain before stretch and the strain at break, respectively. The cyclic tensile and compression tests were conducted with no time interval between two adjacent cycles.

*Single-notch tensile (SNT) test*: The SNT test was carried out to measure the fracture energy of AQGL gel. For the SNT test, two different samples synthesized in the same batch, unnotched and notched (the notch with a length of ~1.5 mm was made in the middle of the sample's right side of the specimen by using a razor) samples with the same dimension were separately stretched to record the force-length curve. The fracture energy (*Γ*) can be calculated *via* the following equation:

$$\Gamma= \frac{U(L_{c})}{a_{0}b_{0}}$$

in which L_c_ is the critical distance (defined as the sample length when the notch starts to propagate) of the notched specimen, U(L_c_) is the work done by the applied force calculated by integrating the force-length curve of the unnotched specimen, and *a*_0_ and *b*_0_ represent the width and thickness of the sample.

*Anti-freezing performances and long-term stability*: To evaluate the anti-freezing performance of the AQ and AQGL gels, the samples were placed in a freezer at -40 °C for 24 h. Then, the samples were taken out and assessed the flexibility by applying deformations with hands. In addition, the samples were placed in the ambient environment for 100 days to evaluate the long-term stability. The weight and electrical resistance of the samples was measured after a fixed time interval during the tests. The weight retention was defined as:

$$Weight retention \left( \% \right)= {W_{t}}/{W_{0} \times100\%}$$

where W_0_ and W_t_ are the initial weight and the weight of the sample at the given storage time, respectively.

*Self-healing performance*: To evaluate the self-healing ability of AQGL gel, a dumbbell-shaped sample was cut into two halves at first. Afterward, these two separated halves were brought into connected and placed at different temperatures for 48 h. After healing, the tensile curves were tested to evaluate the self-healing efficiency.

*Adhesion tests*: The adhesion strength of AQGL organohydrogel to various substrates was measured *via* a 90° peeling tests at a fixed peeling speed of 50 mm/min. The interfacial toughness was determined by dividing the plateau force by sample width.

*Antibacterial tests*: The antibacterial properties of the AQGL gel samples against *S. aureus* (ATCC 6538) and *E. coli* (ATCC 8739) were tested by the Chengdu Ceshigo Research Service Center through the commonly used colony count and zone of inhibition methods, where all the test conditions are based on the current national standard GB/T 20944.3-2008. The average number of the viable bacteria immediately tested after inoculation is represented by U_0_. The initial bacteria suspension concentration of the *S. aureus* is 3.9×10^5^ CFU/mL and that of the *E. coli* is 3.6×10^5^ CFU/mL, which can meet the standard test requirements. After culturing for 18 h, all samples were recovered immediately by adding 10 ml of SCDLP culture solution to the petri dish and rinsing the sample thoroughly, then counting the colonies to evaluate the antibacterial properties. According to the counting results, the average number of the viable S. aureus and E. coli after bacterial culture of the control groups is 3.7×10^7^ CFU/cm^2^ and 5.3×10^6^ CFU/cm^2^, while that of the experiment groups is ＜1.0×10^0^ CFU/cm^2^ and ＜1.0×10^0^ CFU/cm^2^. The antibacterial rate and the antibacterial activity can be calculated as the following equations:

Antibacterial rate (%) = (M-N)/M*100%

Antibacterial activity = U_t_-A_t_

where M is the average number of the surviving bacteria cells of the three control samples on the plates after 18 h culturing, and N is that of the tested three hydrogels, respectively. In addition, U_t_ and A_t_ are the logarithm of M and N respectively. Thus, the related antibacterial properties of the AQGL gel can be calculated. Meanwhile, the inhibition zone diameter of the three control groups and the AQGL gels after 18 h culturing can be directly obtained from the test results.

Cell cultivation: the biocompatibility of the AQGL gel was evaluated using the Cell Counting Kit-8 (CCK-8) assay. Initially, the gel was sterilized by exposure to ultraviolet light for 30 minutes. Subsequently, the sample was placed in a 96-well cell culture plate. MC3T3-L1 cells (GNM15, Shanghai Cell Bank, Chinese Academy of Sciences) in the logarithmic growth phase were seeded onto the hydrogel surface at a density of 4×10^3^ cells per well with 100 μL of phosphate-buffered saline (PBS). The samples were then co-cultured in an incubator maintained at 37 °C and 5% CO_2_. After 1, 4, and 7 days of cultivation, the culture medium was removed, and the cells were washed twice with PBS. Thereafter, 100 μL of CCK-8 reagent (prepared as 90% culture medium and 10% CCK-8) was added under dark conditions. Following a 2-hour incubation period, the absorbance was measured at 450 nm. Cell viability was calculated using the following equation:

Cell viability = (OD (experimental) – OD (background)) / (OD (control) – OD (background)) × 100%

in which the background OD value is the absorbance of the multi-well plate culture itself (the wells with medium but no cells added). For live/dead staining, MC3T3-L1 cells co-cultured for 1, 4, and 7 days were stained with LIVE/DEAD staining solution (containing 2 mM calcein AM and 4 mM propidium iodide) for 30 minutes. Afterwards, the cells were subsequently washed five times with PBS, and images were then captured using an inverted fluorescence microscope (MF53-N, Guangzhou Mingmei Optoelectronics Technology Co., Ltd.).”

*Statistics Analysis:* For the mechanical and multifunctional properties of the AQGL organohydrogel, all experiments were repeated independently with similar results at least 3 times, and the data were expressed as the mean ± standard deviation (SD). For the electrical output signals of the AQGL-TENG, the reported data represent the average value obtained over all measured cycles under a given set of driving conditions.

**S2. Supplemental Tables**

Table S1. Compositions of the A*_x_*Q*_y_*G*_z_*L*_w_* organohydrogels.

|  | **AA (g)** | **CTAB (g)** | **LMA (g)** | **QCS (g)** | **Gly (g)** | **H_2_O (g)** | **Total (g)^a)^** | **LiCl (g)** |
| --- | --- | --- | --- | --- | --- | --- | --- | --- |
| **A_17.5_Q_0.1_G_11_L_0.5_** | 3.50 | 0.42 | 0.28 | 0.10 | 7.85 | 7.85 | 20.00 | 0.30 |
| **A_20.0_Q_0.1_G_11_L_0.5_** | 4.00 | 0.48 | 0.32 | 0.10 | 7.55 | 7.55 | 20.00 | 0.30 |
| **A_22.5_Q_0.1_G_11_L_0.5_** | 4.50 | 0.54 | 0.36 | 0.10 | 7.25 | 7.25 | 20.00 | 0.30 |
| **A_25.0_Q_0.1_G_11_L_0.5_** | 5.00 | 0.60 | 0.40 | 0.10 | 6.95 | 6.95 | 20.00 | 0.30 |
| **A_22.5_Q_0_G_11_L_0.5_** | 4.50 | 0.54 | 0.36 | 0.00 | 7.30 | 7.30 | 20.00 | 0.30 |
| **A_22.5_Q_0.2_G_11_L_0.5_** | 4.50 | 0.54 | 0.36 | 0.20 | 7.20 | 7.20 | 20.00 | 0.30 |
| **A_22.5_Q_0.3_G_11_L_0.5_** | 4.50 | 0.54 | 0.36 | 0.30 | 7.15 | 7.15 | 20.00 | 0.30 |
| **A_22.5_Q_0.2_G_12_L_0.5_** | 4.50 | 0.54 | 0.36 | 0.20 | 4.80 | 9.60 | 20.00 | 0.30 |
| **A_22.5_Q_0.2_G_23_L_0.5_** | 4.50 | 0.54 | 0.36 | 0.20 | 5.75 | 8.65 | 20.00 | 0.30 |
| **A_22.5_Q_0.2_G_32_L_0.5_** | 4.50 | 0.54 | 0.36 | 0.20 | 8.65 | 5.75 | 20.00 | 0.30 |
| **A_22.5_Q_0.2_G_21_L_0.5_** | 4.50 | 0.54 | 0.36 | 0.20 | 9.60 | 4.80 | 20.00 | 0.30 |
| **A_22.5_Q_0.2_G_11_L_0_** | 4.50 | 0.54 | 0.36 | 0.20 | 7.20 | 7.20 | 20.00 | 0.00 |
| **A_22.5_Q_0.2_G_11_L_0.25_** | 4.50 | 0.54 | 0.36 | 0.20 | 7.20 | 7.20 | 20.00 | 0.15 |
| **A_22.5_Q_0.2_G_11_L_0.75_** | 4.50 | 0.54 | 0.36 | 0.20 | 7.20 | 7.20 | 20.00 | 0.45 |
| **A_22.5_Q_0.2_G_11_L_1.0_** | 4.50 | 0.54 | 0.36 | 0.20 | 7.20 | 7.20 | 20.00 | 0.60 |

Note: ^a)^ The total mass does not include the mass of LiCl.

Table S2. The mechanical properties of A*_x_*Q_0.1_G_11_L_0.5_ organohydrogels with different AA contents.

|  | **A_17.5_Q_0.1_G_11_L_0.5_** | **A_20.0_Q_0.1_G_11_L_0.5_** | **A_22.5_Q_0.1_G_11_L_0.5_** | **A_25.0_Q_0.1_G_11_L_0.5_** |
| --- | --- | --- | --- | --- |
| **σ_t_ (MPa)^a)^** | \| 0.061 ± 0.006 \| \| --- \| | 0.202 ± 0.012 | 0.299 ± 0.010 | 0.506 ± 0.050 |
| **ε (%)^b)^** | 2650 ± 427 | 2471 ± 188 | 4696 ± 450 | 2641 ± 368 |
| **E_t_ (kPa)^c)^** | 4.7 ± 0.9 | 7.1 ± 0.3 | 9.8 ± 0.1 | 22.5 ± 1.6 |
| **T (MJ/m^3^)^d)^** | 0.85 ± 0.06 | 2.89 ± 0.39 | 8.78 ± 1.46 | 6.79 ± 1.76 |
| **σ_c_ (MPa)^e)^** | 0.85 ± 0.03 | 1.23 ± 0.17 | 1.52 ± 0.15 | 2.92 ± 0.24 |
| **E_c_ (kPa)^f)^** | 14.8 ± 1.4 | 26.6 ± 3.2 | 37.9 ± 2.1 | 72.4 ± 5.1 |

Note: ^a)^ tensile stress; ^b)^ tensile strain; ^c)^ Young's modulus; ^d)^ toughness; ^e)^ compression stress; ^f)^ compression modulus.

Table S3. The mechanical properties of A_22.5_Q*_y_*G_11_L_0.5_ organohydrogels with different QCS contents.

|  | **A_22.5_Q_0_G_11_L_0.5_** | **A_22.5_Q_0.1_G_11_L_0.5_** | **A_22.5_Q_0.2_G_11_L_0.5_** | **A_22.5_Q_0.3_G_11_L_0.5_** |
| --- | --- | --- | --- | --- |
| **σ_t_ (MPa)** | 0.258 ± 0.020 | 0.299 ± 0.010 | 0.331 ± 0.013 | 0.339 ± 0.022 |
| **ε (%)** | 3488 ± 437 | 4696 ± 450 | 5800 ± 330 | 3696 ± 279 |
| **E_t_ (kPa)** | 9.2 ± 0.74 | 9.8 ± 0.1 | 14.3 ± 0.5 | 12.7 ± 0.8 |
| **T (MJ/m^3^)** | 5.40 ± 1.09 | 8.78 ± 1.46 | 11.77 ± 0.87 | 7.02 ± 1.17 |
| **σ_c_ (MPa)** | 1.22 ± 0.12 | 1.52 ± 0.15 | 1.62 ± 0.09 | 1.80 ± 0.10 |
| **E_c_ (kPa)** | 16.0 ± 2.7 | 32.7 ± 4.0 | 37.2 ± 3.3 | 59.5 ± 5.2 |

Table S4. The mechanical properties of A_22.5_Q_0.2_G*_z_*L_0.5_ organohydrogels with different Gly/H_2_O ratios.

|  | **A_22.5_Q_0.2_L_0.5_** | **A_22.5_Q_0.2_G_12_L_0.5_** | **A_22.5_Q_0.2_G_23_L_0.5_** | **A_22.5_Q_0.2_G_11_L_0.5_** | **A_22.5_Q_0.2_G_32_L_0.5_** | **A_22.5_Q_0.2_G_21_L_0.5_** |
| --- | --- | --- | --- | --- | --- | --- |
| **σ_t_ (MPa)** | 0.780 ± 0.022 | 0.563 ± 0.036 | 0.441 ± 0.035 | 0.331 ± 0.013 | 0.314 ± 0.013 | 0.242 ± 0.021 |
| **ε (%)** | 1119 ± 445 | 2606 ± 295 | 3859 ± 317 | 5800 ± 330 | 3990 ± 667 | 4285 ± 398 |
| **E_t_ (kPa)** | 70.0 ± 13.1 | 28.1 ± 4.2 | 17.6 ± 1.6 | 14.3 ± 0.5 | 12.3 ± 1.2 | 9.0 ± 0.5 |
| **T (MJ/m^3^)** | 4.20 ± 0.17 | 8.22 ± 1.09 | 10.43 ± 1.40 | 11.77 ± 0.87 | 7.62 ± 1.89 | 6.34 ± 1.23 |
| **σ_c_ (MPa)** | 5.01 ± 0.24 | 3.02 ± 0.18 | 2.42 ± 0.35 | 1.62 ± 0.09 | 0.95 ± 0.08 | 0.65 ± 0.12 |
| **E_c_ (kPa)** | 98.5 ± 8.2 | 79.6 ± 6.2 | 52.0 ± 2.1 | 37.2 ± 3.3 | 43.1 ± 7.4 | 27.4 ± 2.9 |

Table S5. The mechanical properties of A_22.5_Q_0.2_G_11_L*_w_* organohydrogels with different LiCl concentration.

|  | **A_22.5_Q_0.2_G_11_L_0_** | **A_22.5_Q_0.2_G_11_L_0.25_** | **A_22.5_Q_0.2_G_11_L_0.5_** | **A_22.5_Q_0.2_G_11_L_0.75_** | **A_22.5_Q_0.2_G_11_L_1.0_** |
| --- | --- | --- | --- | --- | --- |
| **σ_t_ (MPa)** | 0.185 ± 0.025 | 0.221 ± 0.018 | 0.331 ± 0.013 | 0.333 ± 0.036 | 0.420 ± 0.039 |
| **ε (%)** | 4900 ± 559 | 5240 ± 215 | 5800 ± 330 | 3810 ± 438 | 3373 ± 240 |
| **E_t_ (KPa)** | 5.8 ± 0.8 | 8.3 ± 0.7 | 14.3 ± 0.5 | 17.8 ± 1.1 | 25.0 ± 2.1 |
| **T (MJ/m^3^)** | 5.59 ± 1.43 | 6.75 ± 0.82 | 11.77 ± 0.872 | 6.05 ± 1.43 | 7.80 ± 1.44 |
| **σ_c_ (MPa)** | 0.47 ± 0.07 | 0.89 ± 0.13 | 1.62 ± 0.09 | 2.48 ± 0.20 | 2.98 ± 0.17 |
| **E_c_ (kPa)** | 14.3 ± 1.8 | 21.7 ± 2.4 | 37.2 ± 3.3 | 51.2 ±3.5 | 84.2 ± 8.0 |

Table S6. Summary of mechanical properties of recently reported tough gels.

| **Label** | **Reference** | **Mechanical property** | | | | | **Functionality** | | | |
| --- | --- | --- | --- | --- | --- | --- | --- | --- | --- | --- |
|  |  | **σ_t_**  **(MPa)** | **ε**  **(%)** | **E**  **(kPa)** | **T**  **(MJ/m^3^)** | **Г**  **(kJ/m^2^)** | **Self-**  **healing** | **Anti-**  **freezing** | **Transparent** | **Adhesive** |
|  | **This work** | **0.331** | **5800** | **14.3** | **11.77** | **75.96** | √ | √ | √ | √ |
| S1 | *Nature* **2019**, 575, 169 | 0.13 | 700 | 15 | -- | 1.70 | -- | -- | -- | √ |
| S2 | *Nat. Mater.*, **2020**, 19, 1102 | ~0.50 | 283 | 100 | -- | 0.12 | √ | √ | -- | √ |
| S3 | *Sci. Adv.* **2024***,* 10, eadj5389 | 0.12 | 696 | 19.1 | ~0.05 | -- | √ | -- | √ | √ |
| S4 | *Joule* **2021**, 5, 2211 | 1.19 | 217 | 150 | 2.85 | 2.77 | -- | -- | -- | -- |
| S5 | *Matter* **2020***,* 3*,* 1196 | 0.12 | 150 | 120 | 0.10 | -- | -- | -- | -- | -- |
| S6 | *Adv. Mater.* **2024***,* 2309508 | 0.22 | 900 | 60 | 1.02 | -- | -- | √ | √ | -- |
| S7 | *Adv. Mater*., **2021**, 2101500 | ~0.06 | 950 | 18.0 | -- | 0.80 | -- | -- | -- | -- |
| S8 | *ACS Nano,* **2024***,* 18*,* 3720 | ~2.20 | 780 | 873 | 8.23 | -- | -- | √ | -- | -- |
| S9 | *Nano-Micro. Lett.,* **2023**, 15, 8 | 0.19 | 1586 | 43.3 | 1.30 | -- | -- | -- | √ | √ |
| S10 | *Adv. Funct. Mater.,* **2024***,* 2314864 | 0.19 | 1153 | 95.2 | 1.18 | -- | -- | √ | √ | -- |
| S11 | *Adv. Funct. Mater.,* **2023***,* 2301127 | ~0.10 | 1224 | 30 | 0.56 | -- | -- | -- | -- | √ |
| S12 | *Adv. Funct. Mater.* **2023***,* 2211027 | 0.05 | 520 | 20 | 0.11 |  | -- | √ | √ | -- |
| S13 | *Adv. Funct. Mater.,* **2021***,* 2103117 | 1.30 | 1400 | 110 | 2.98 | -- | -- | -- | √ | -- |
| S14 | *Adv. Funct. Mater*., **2021**, 2101095 | 2.25 | 340 | 3400 | -- | 2.00 | -- | -- | -- | -- |
| S15 | *Adv. Funct. Mater.,* **2021***,* 2011176 | 0.36 | 480 | 80 | 0.94 | -- | -- | √ | -- | -- |
| S16 | *Adv. Funct. Mater.,* **2021***,* 2106761 | 0.15 | 4000 | 40 | 0.50 | -- | √ | -- | -- | √ |
| S17 | *Nano Energy,* **2022***,* 95, 106967 | 0.38 | 300 | 123.5 | 0.40 | -- | √ | √ | √ | -- |
| S18 | *Nano Energy,* **2022***,* 100, 107438 | 1.0 | 1300 | 42 | -- | 13.46 | √ | √ | √ | -- |
| S19 | *Nano Energy,* **2022**, 99, 107374 | 0.12 | 1300 | 1550 | ~1.50 | -- | √ | √ | -- | -- |
| S20 | *Mater. Horiz.,* **2021***,* 8*,* 1795 | 0.9 | 2590 | 52.3 | 7.85 | 4.20 | √ | -- | -- | -- |
| S21 | *Chem. Mater.,* **2022***,* 34, 5258 | 0.18 | 950 | 38.5 | 0.08 | ~0.09 | -- | -- | -- | √ |
| S22 | *Chem. Mater.,* **2022***,* 34*,* 1392 | 1.38 | 2150 | 128 | 15.66 | 13.99 | √ | √ | -- | -- |
| S23 | *Chem. Eng. J.,* **2022***,* 431, 133782 | 0.08 | 1095 | ~80 | 0.05 | -- | -- | √ | -- | √ |
| S24 | *Chem. Eng. J.,* **2023***,* 477, 147065 | 1.324 | 1250 | 365 | 9.00 | -- | -- | √ | -- | -- |
| S25 | *Chem. Eng. J.,* **2023***, 466,* 143087 | 0.40 | 1324 | 77.8 | 3.07 | 3.15 | √ | √ | √ | √ |
| S26 | *Carbohyd Polym.*, **2024***, 328,* 121728 | 0.12 | 2100 | 680 | 1.30 | -- | -- | √ | -- | -- |
| S27 | *J. Mater. Chem. A,* **2023***,* 11*,* 24608 | 0.17 | 1610 | 400 | 1.35 | -- | √ | √ | -- | √ |
| S28 | *J. Mater. Chem. A,* **2024***,* 12, 3589 | ~0.03 | 639 | 27.1 | 1.37 | -- | √ | √ | √ | √ |
| S29 | *Nat. Commun*., **2023**, 14, 130* |  | 1542 | 830 | -- | 111.16 | √ | -- | -- | -- |
| S30 | *Nat. Commun*., **2020**, 11, 1107* | 2.42 | 510 | 640 | -- | 3.67 | -- | √ | -- | -- |

Note: “Г” denotes fracture energy, while the symbol “--” means not mentioned in the literature and the superscript “*” represents that the material is an elastomer.

Table S7. Test results of the antibacterial properties against *S. aureus* and *E. coli*.

| Tested bacteria | U_0_  (CFU/cm^2^) | M  (CFU/cm^2^) | N  (CFU/cm^2^) | Antibacterial  rate (%) | Antibacterial activity | Inhibition zone diameter (mm) |
| --- | --- | --- | --- | --- | --- | --- |
| *S. aureus* | 3.9×10^5^ | 3.7×10^7^ | ＜1.0×10^0^ | ＞99.9999 | 7.57 | 14.4 |
| *E. coli* | 3.6×10^5^ | 5.3×10^6^ | ＜1.0×10^0^ | ＞99.9999 | 6.72 | 8.3 |

Table S8. Summary of mechanical properties, electrical conductivity and TENG output performance of recently reported gel systems for TENG applications.

| **Reference** | **Tensile strain (%)** | **Tensile stress (MPa)** | **Modulus (kPa)** | **Toughness (MJ/m^3^)** | **Conductivity (S/m)** | **Power density (W/m^2^)** |
| --- | --- | --- | --- | --- | --- | --- |
| This work | 5800 | 0.331 | 14.3 | 11.77 | 0.760 | 0.960 |
| *Matter* **2023**, 6, 1514 | ~1200 | ~0.400 | --- | -- | 0.574 | ~0.150 |
| *Adv. Funct. Mater*. **2025**, 35, 241164 | ~472 | 0.212 | 45.3 | ~0.38 | 0.060 | 0.464 |
| *Adv. Funct. Mater*. **2025**, 35, 2501362 | 349 | 0.283 | 129 | ~0.48 | ~0.210 | 0.462 |
| *Adv. Funct. Mater*. **2025**, e22340 | 1375 | ~0.225 | --- | ~1.49 | 2.60 | 1.250 |
| *Nano Energy* **2024**, 127, 109772 | ~566 | ~0.090 | --- | --- | 12.6 | 0.510 |
| *Small* **2025**, e12928 | 1082 | ~0.451 | ~38.9 | 2.64 | ~1.15 | 0.31*10^-3^ |
| *Adv. Funct. Mater.* **2024***, 2414682* | 708 | 5.9 | 30 | 22 | 0.64 | 2.46 |
| *Chem. Enm. J.* **2025***, 511, 161803* | --- | --- | --- | --- | 0.00194 | ~0.45 |
| *Adv. Funct. Mater.* **2025***, 2505265* | 3501 | 1.21 | -- | 12.87 | 0.22 | 0.205 |
| *ACS Appl. Mater. Interfaces* **2025***,* *17, 52738* | 67 | 5.71 | -- | 2.43 | --- | --- |

Table S9. Summary of the output performance of AQGL-TENG with other TENGs reported in literature.

| **Reference** | **Triboelectric materials/electrode** | | **Driven conditions** | **Voltage (V)** | **Current (μA)** | **Power density (W/m^2^)** | **Electrode multifunctionality** | |
| --- | --- | --- | --- | --- | --- | --- | --- | --- |
| This work | Ecoflex/AQGL organohydrogel | | 10 N, 1 Hz | 352 | 4.5 | 0.960 | Self-Healing, anti-freezing, transparent, adhesive | |
| *Adv. Funct. Mater.* **2025***, 2505265* | Hydrogel/PTFE/Cu | | --- | 127 | 1.5 | 0.205 | --- | |
| *Adv. Mater.* **2025***, 39 2510431* | PTFE/LCP/Cu | | 2 Hz | 73.6 | --- | 1.07 | --- | |
| *ACS Appl. Mater. Interfaces* **2025***,* *17, 19054* | Nylon/PTFE/Cu | | --- | 140 | 1.2 | --- | --- | |
| *Small* **2025**, e12928 | PTFE/PAM hydrogel @ ZnSnO_3_ QDs | | 20 N, 1 Hz | 35.6 | ＞0.2 | 0.31*10^-3^ | --- | |
| *Chem. Eng. J.* **2025***, 511, 161803* | Ecoflex/PTC-1 hydrogel | | 40 N/4 Hz | 255 | 4.2 | ~0.45 | Anti-freezing | |
| *Chem. Eng. J.* **2024***, 499, 156650* | Ecoflex/ PEO@LiTFSI gel | --- | | 105 | 9.2 | 0.36 | | Adhesive |
| *Adv. Funct. Mater.* **2021**, 2104928 | Ecoflex/Mxene@PVA hydrogel | | --- | 230 | 0.27 | 0.33 | --- | |

**S3. Supplemental Figures**


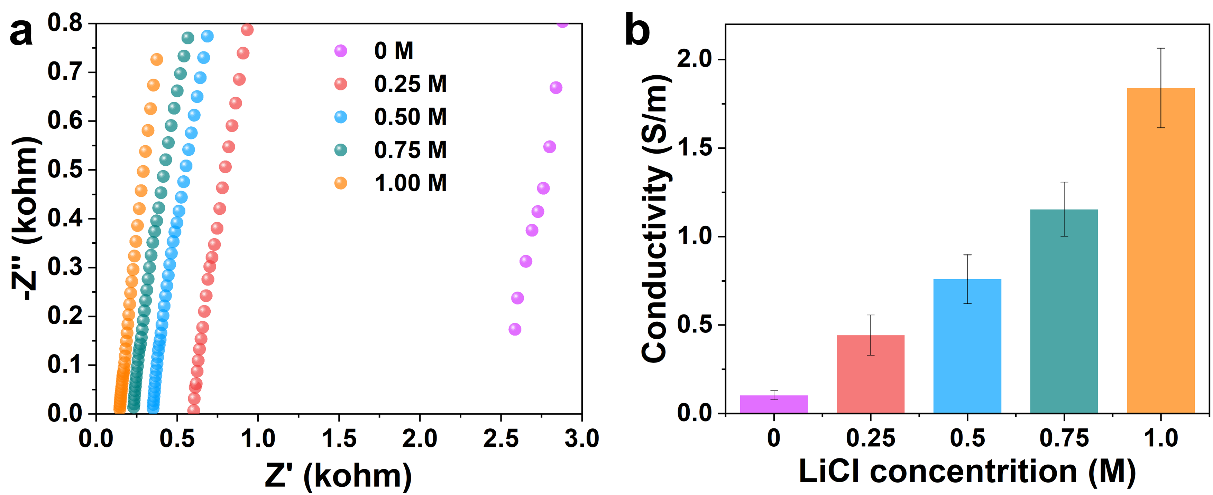


Figure S1. The EIS Nyquist plot of A_22.5_Q_0.2_G_11_L*_w_* organohydrogels with different LiCl concentrations.


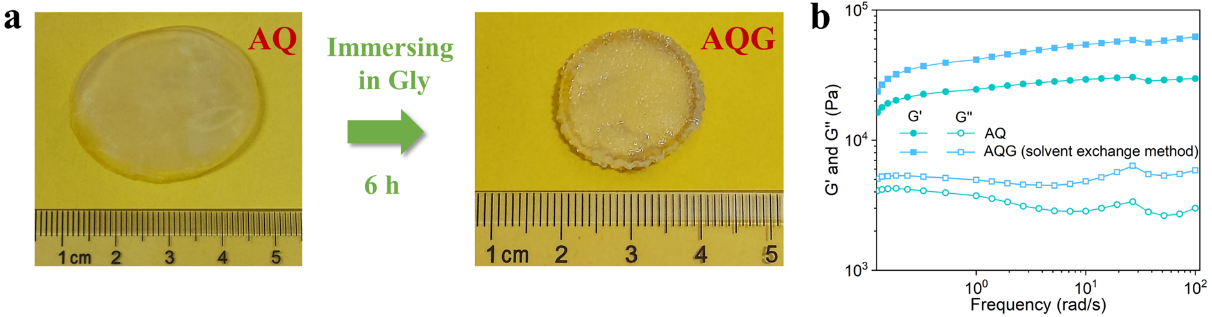


Figure S2. (a) The pictures show the obvious shrinkage of the AQ hydrogel after immersing in glycerol for 6 hours. (b) Dynamic rheology analysis shows the higher storage (G’) and loss modulus (G’’) of AQG organohydrogel (prepared by solvent exchange method) than the AQ hydrogel.


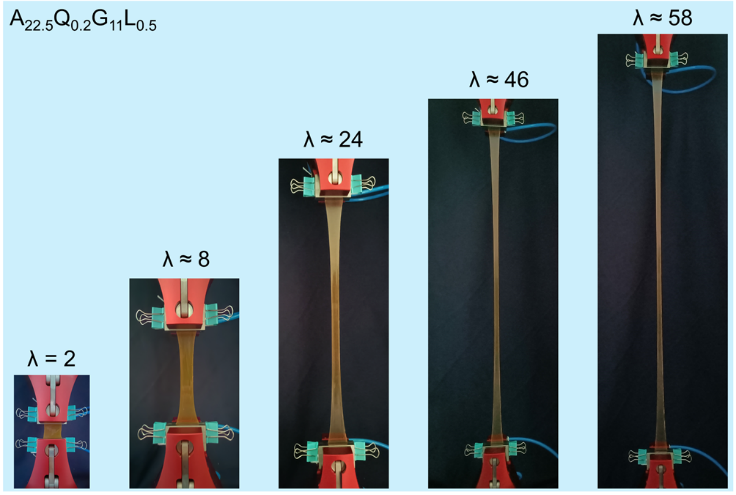


Figure S3. Photographs show the high stretchability of the A_12.5_Q_0.2_G_11_L_0.5_ organohydrogel. For a better visualization, the gel was dyed with yellow pigment.


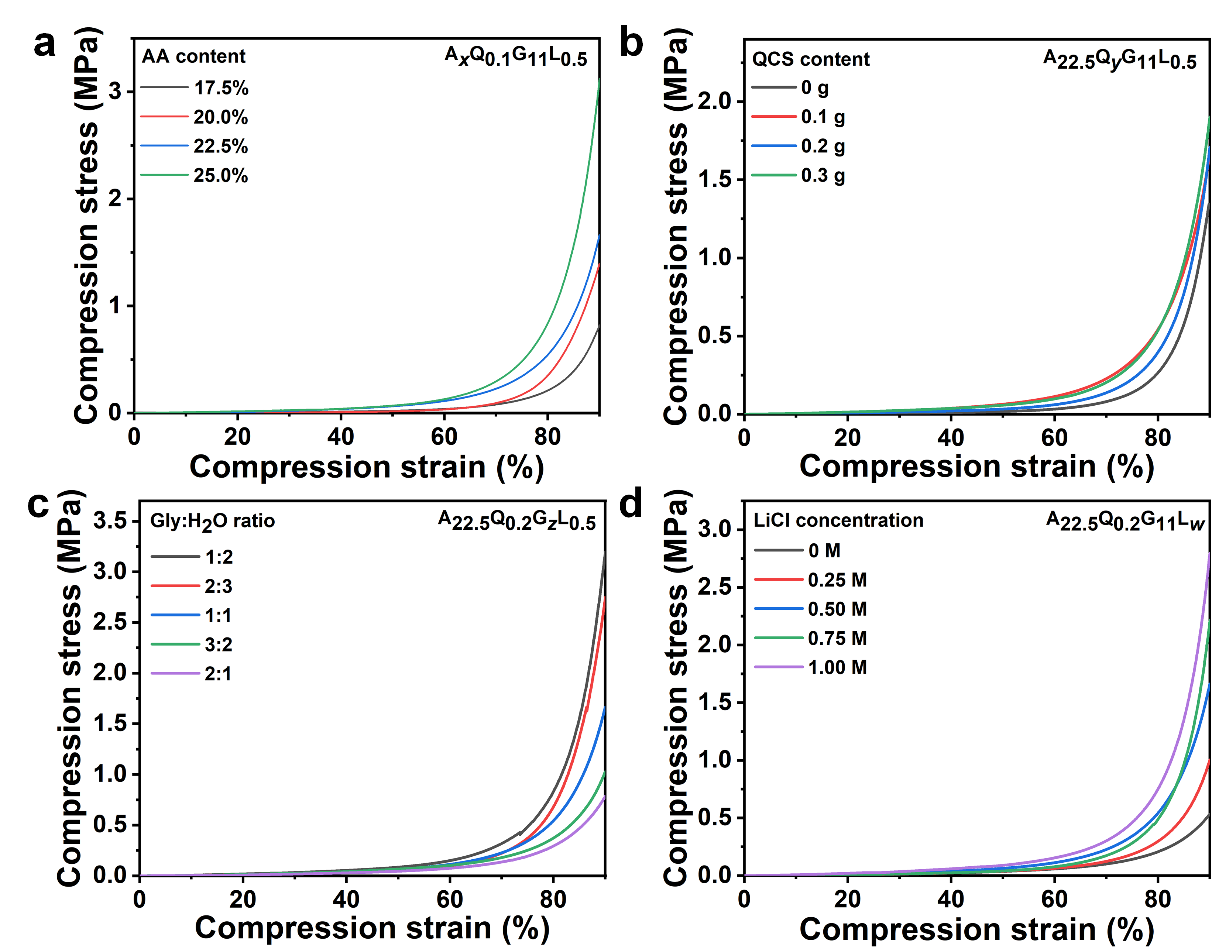


Figure S4. The compression stress-strain curves of (a) A*_x_*Q_0.2_G_11_L_0.5_, (b) A_22.5_Q*_y_*G_11_L_0.5_, (c) A_22.5_Q_0.2_G*_z_*L_0.5_, and (d) A_22.5_Q_0.2_G_11_L_w_ organohydrogels.


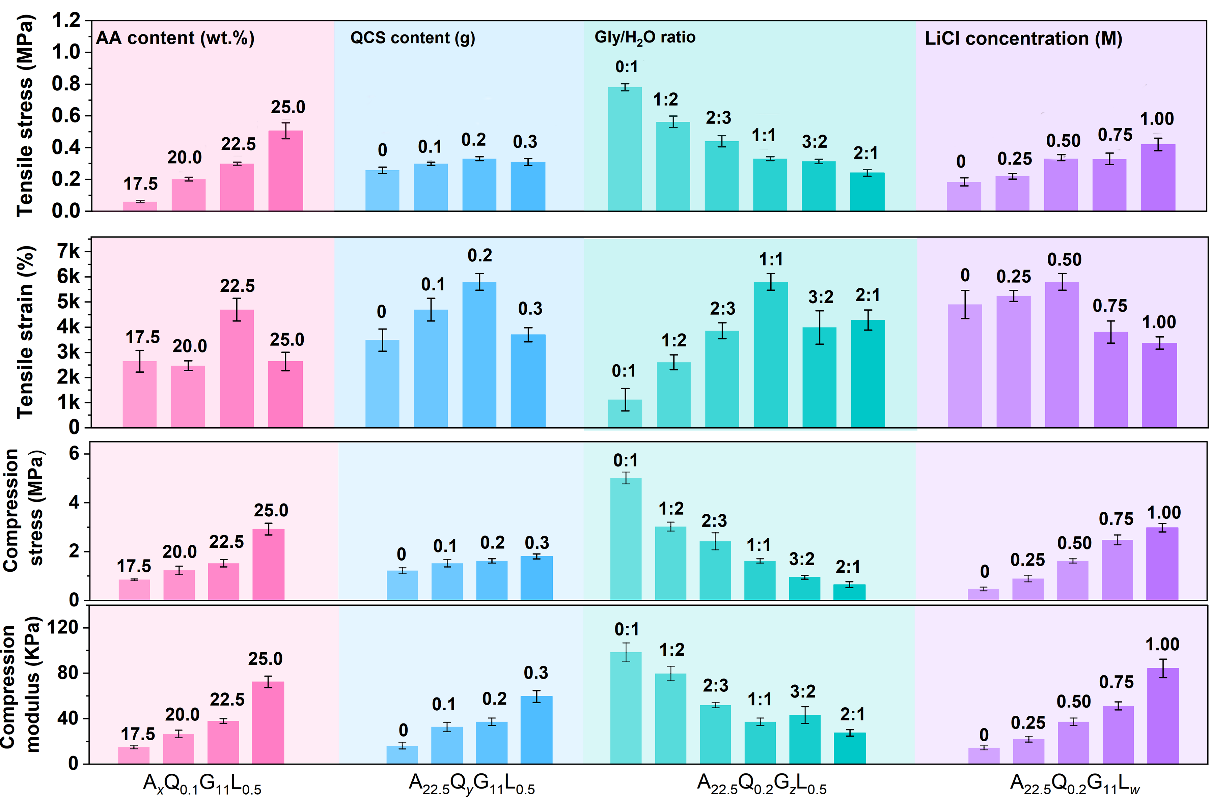


Figure S5. Summary of the mechanical property parameters (tensile stress, tensile strain, compression stress, and compression modulus) of A*_x_*Q_0.2_G_11_L_0.5_, A_22.5_Q*_y_*G_11_L_0.5_, A_22.5_Q_0.2_G*_z_*L_0.5_, and A_22.5_Q_0.2_G_11_L_w_ organohydrogels.


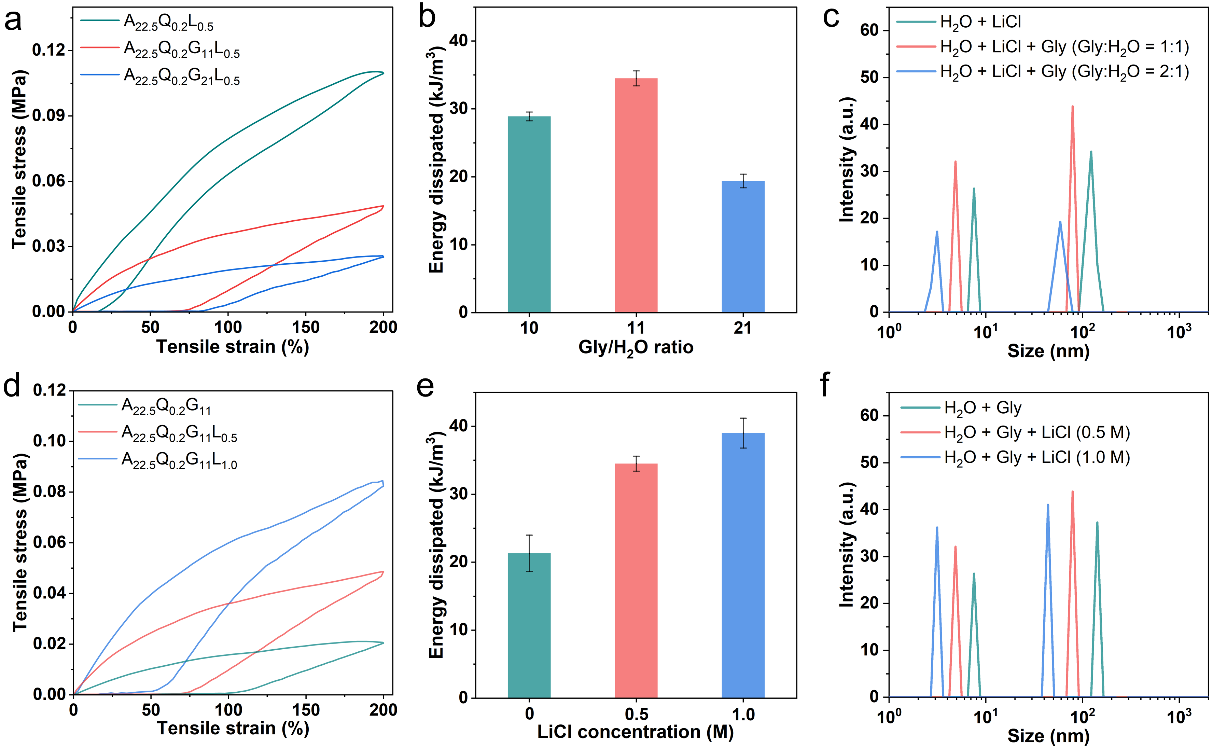


Figure S6. (a) The representative cyclic tensile loading-unloading curves of A_22.5_Q_0.2_G*_z_*L_0.5_ gels with different Gly/H_2_O ratios, and (b) the corresponding dissipated deformation energy calculated from the hysteresis loops. (c) The size distribution of micelles in the precursor solution of A_22.5_Q_0.2_L_0.5_, A_22.5_Q_0.2_G_11_L_0.5_, A_22.5_Q_0.2_G_21_L_0.5_ gels. (d) The representative cyclic tensile loading-unloading curves of A_22.5_Q_0.2_G_11_L*_w_* gels with different LiCl concentrations, and (e) the corresponding dissipated deformation energy calculated from the hysteresis loops. (f) The size distribution of micelles in the precursor solution of A_22.5_Q_0.2_G_11_, A_22.5_Q_0.2_G_11_L_0.5_, A_22.5_Q_0.2_G_11_L_1.0_ gels.


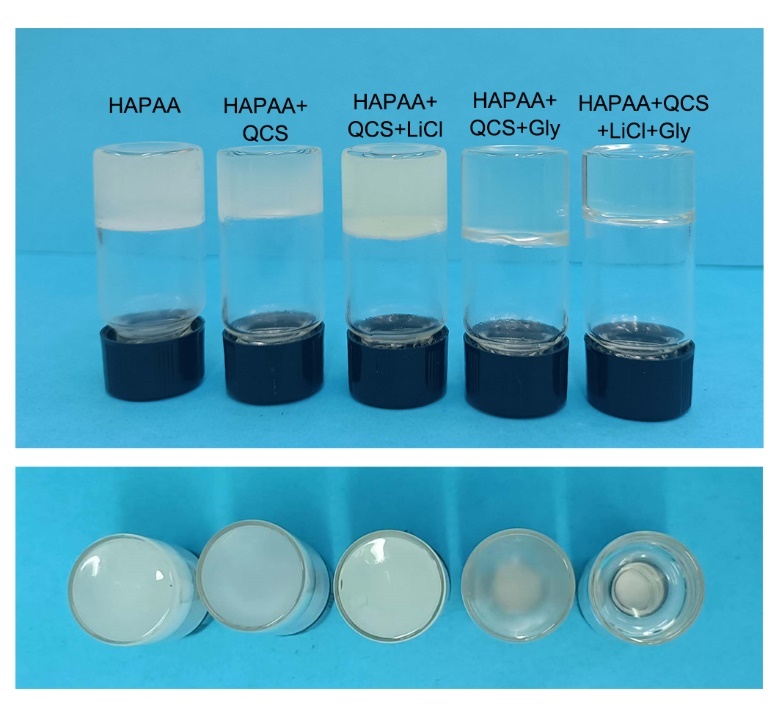


Figure S7. Photographs show the transparency of gels with different compositions.


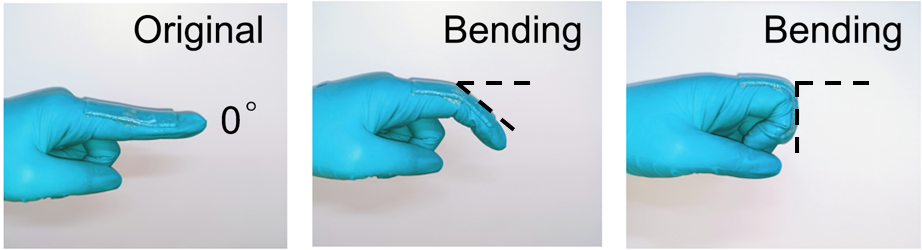


Figure S8. Photographs show AQGL organohydrogel conformally self-adhered to the frequently moving finger joint.


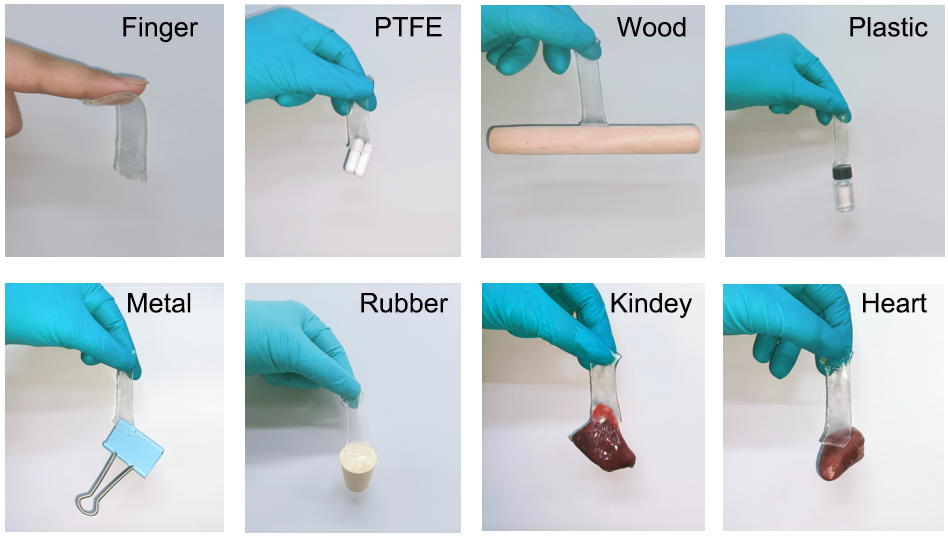


Figure S9. Photographs show the AQGL organohydrogel adhered to diverse organic, inorganic materials and wet tissues.


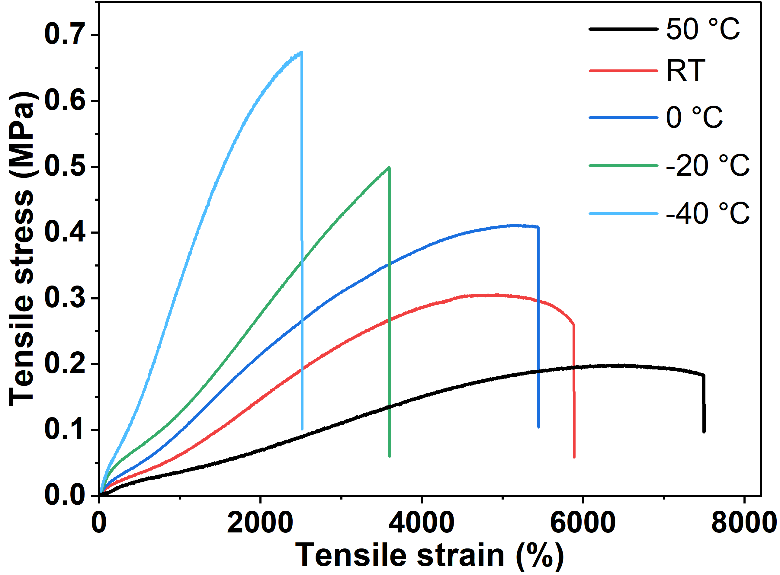


Figure S10. Representative tensile stress-strain curves of the A_22.5_Q_0.2_G_11_L_0.5_ organohydrogel at different temperatures.

**
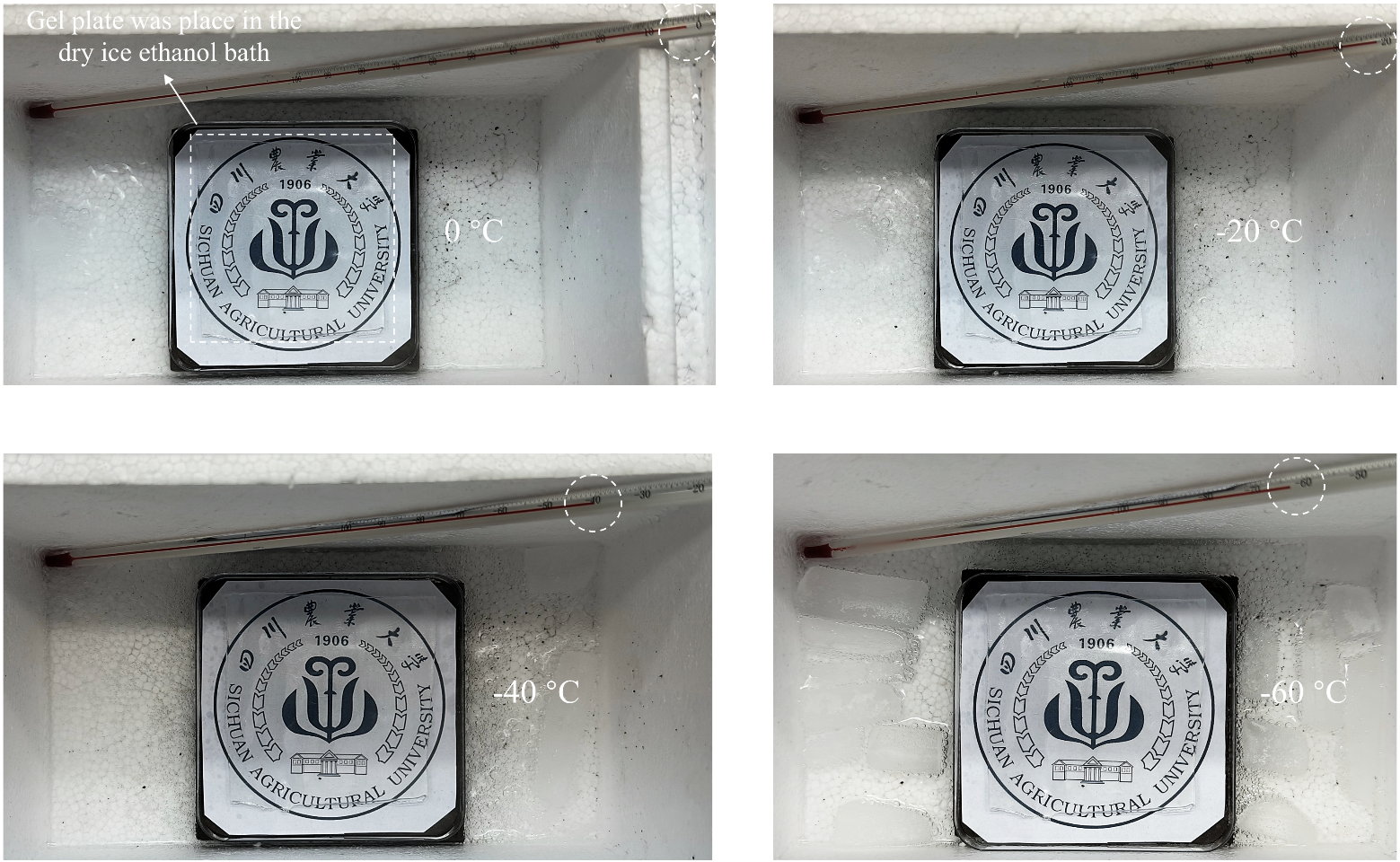
**

Figure S11. Photograph show that the AQGL gel can maintain its high transparency even the temperature was down to -60 °C. The gel plate was placed in a plastic culture dish and directly immersed into the dry ice ethanol bath.


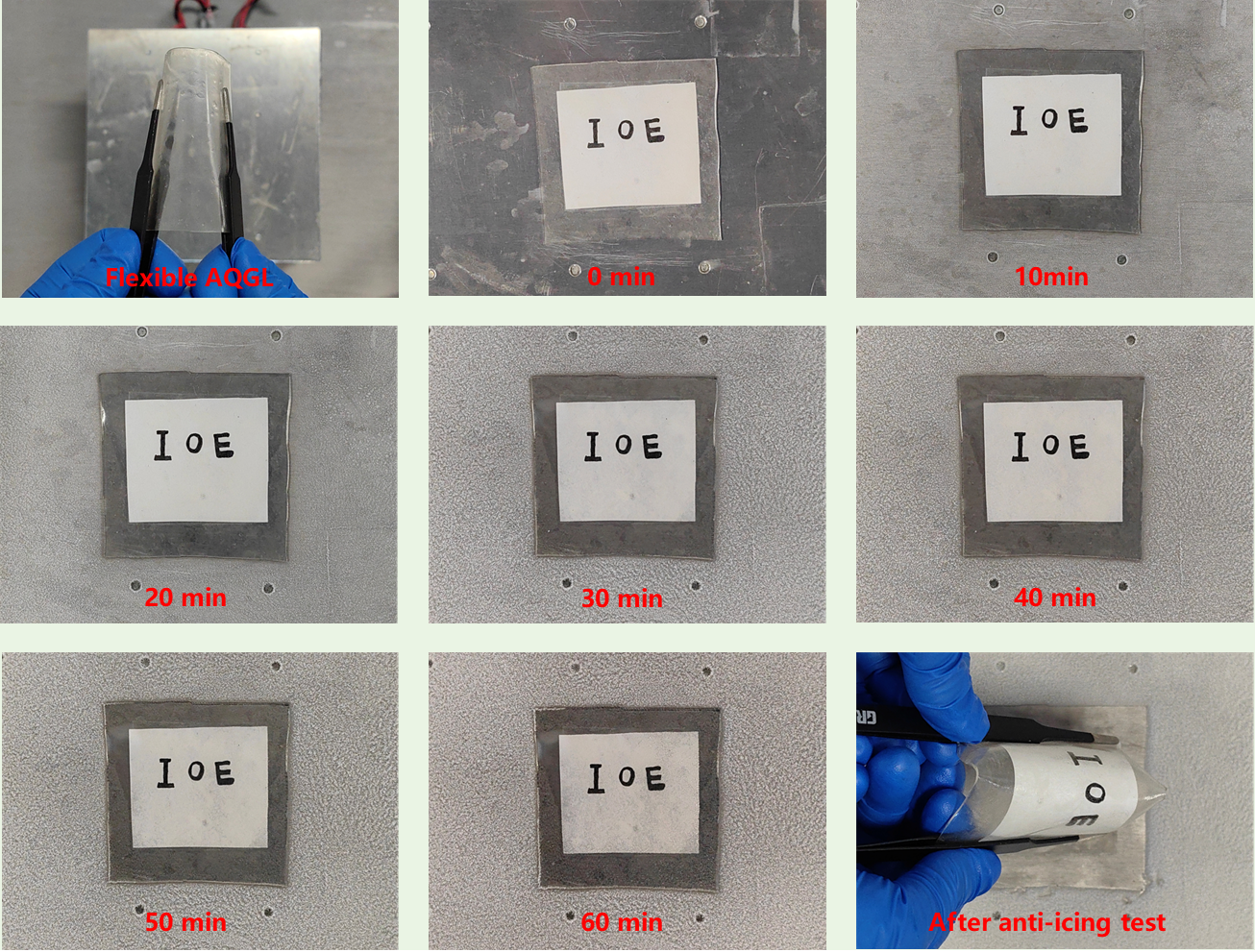


Figure S12. Anti-icing test of AQGL using a thermoelectric cooler at a constant temperature of –15 °C.


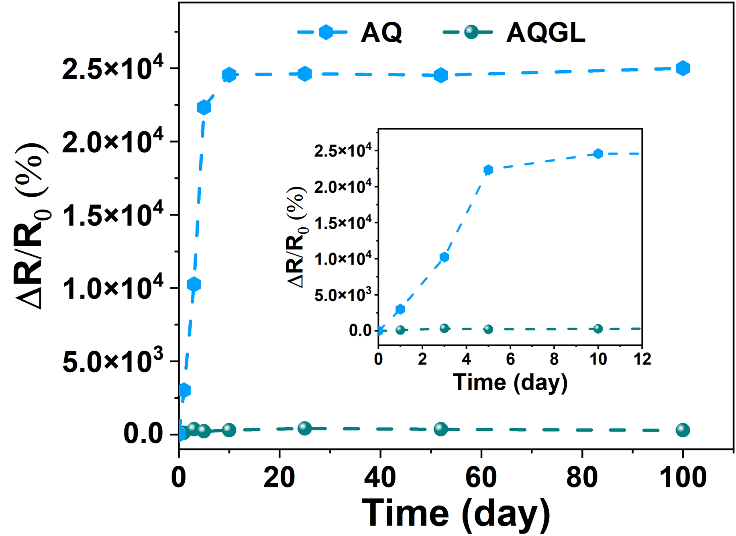


Figure S13. The electrical resistance change ratio of the A_22.5_Q_0.2_ and A_22.5_Q_0.2_G_11_L_0.5_ gels as a function of time upon placing at ambient condition for 100 days.


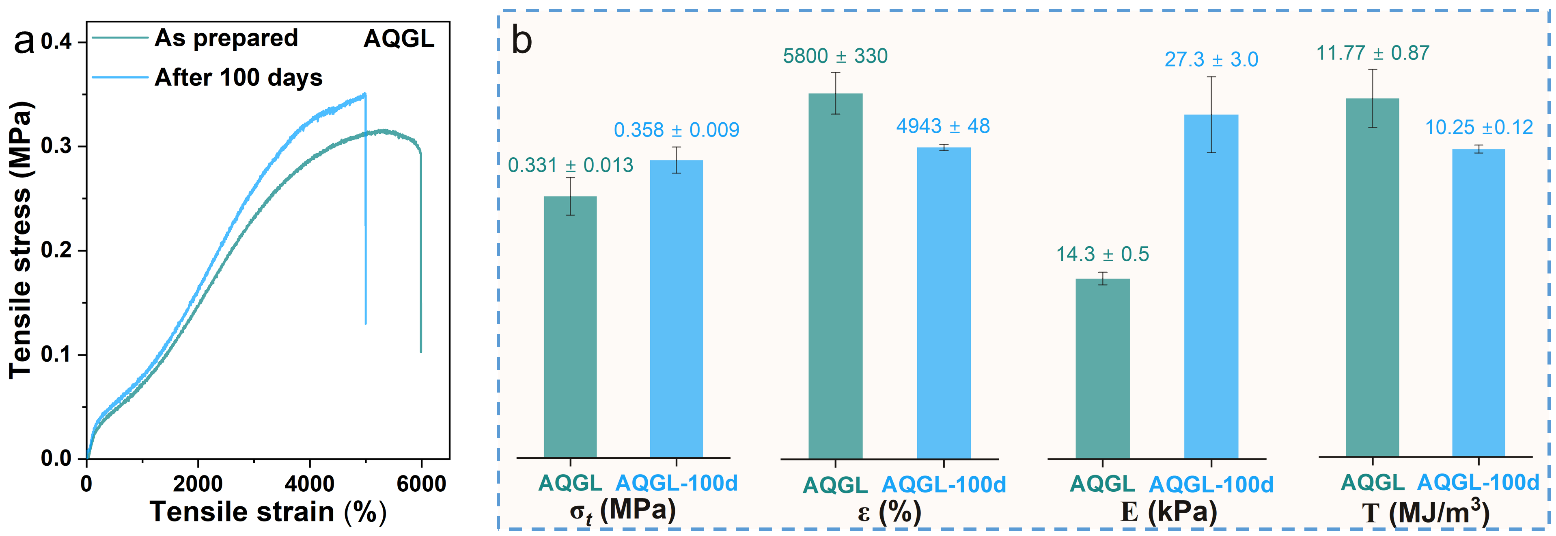


Figure S14. (a) Representative tensile stress-strain curves of the AQGL organohydrogels in its as-prepared state and after 100 days of storage under ambient conditions (denoted as AQGL-100d). (b) Comparison for mechanical parameters of AQGL and AQGL-100d gels.


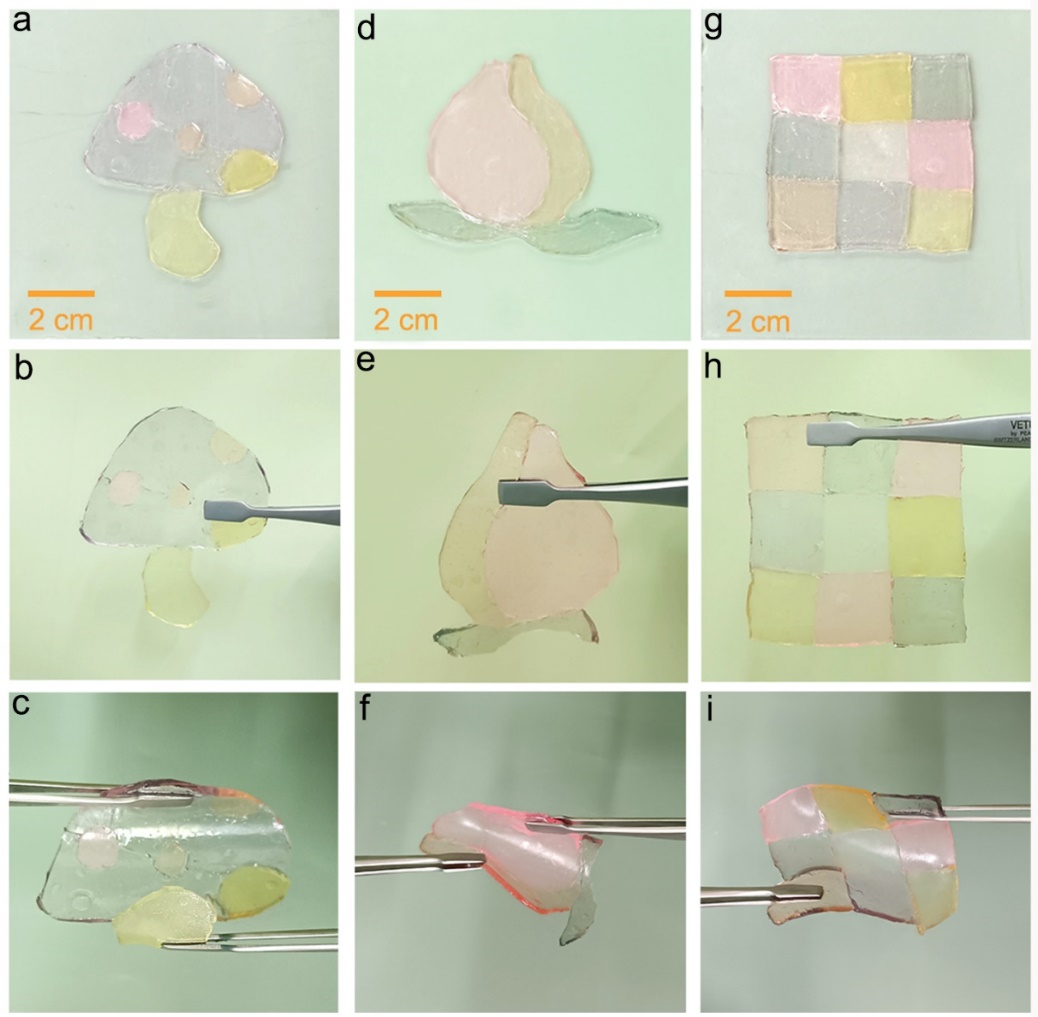


Figure S15. Photographs vividly show the self-healing property of the AQGL organohydrogel. To enhance visualization, AQGL gel were dyed with diverse pigments. Putting the different parts together to form a (a-c) mushroom, (d-f) peach, and (g-i) the surface of a Rubik’s cube. After 1 hour healing at room temperature, all parts were merged into an integrated one that were bendable and twistable.


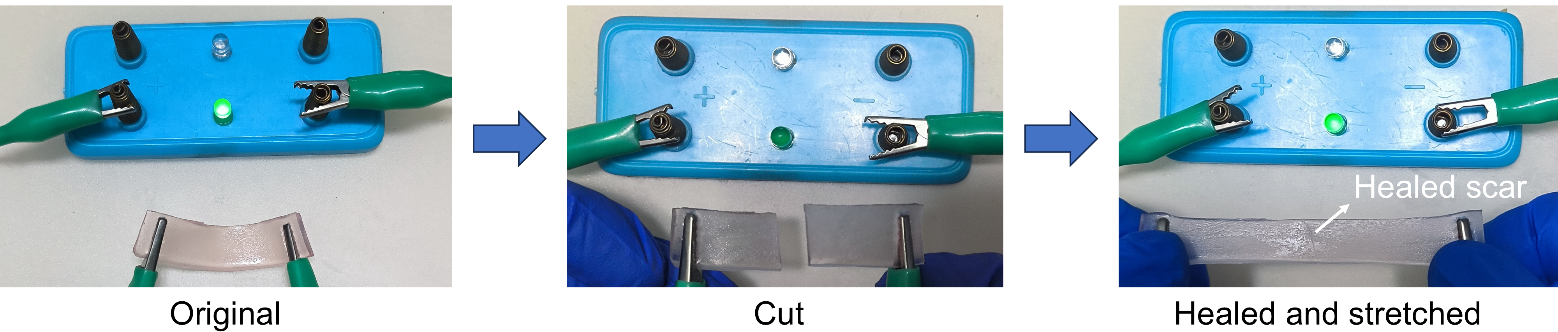


Figure S16. Photographs show the recovery of the electrical conductivity and stretchability of the AQGL organohydrogel after self-healing. The organohydrogel was dyed with light pink color to enhance visualization.


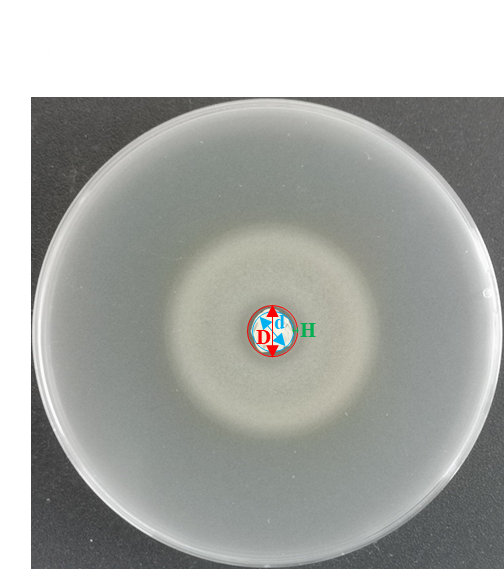


Figure S17. Corresponding inhibition zone diameter against *E. coli.*


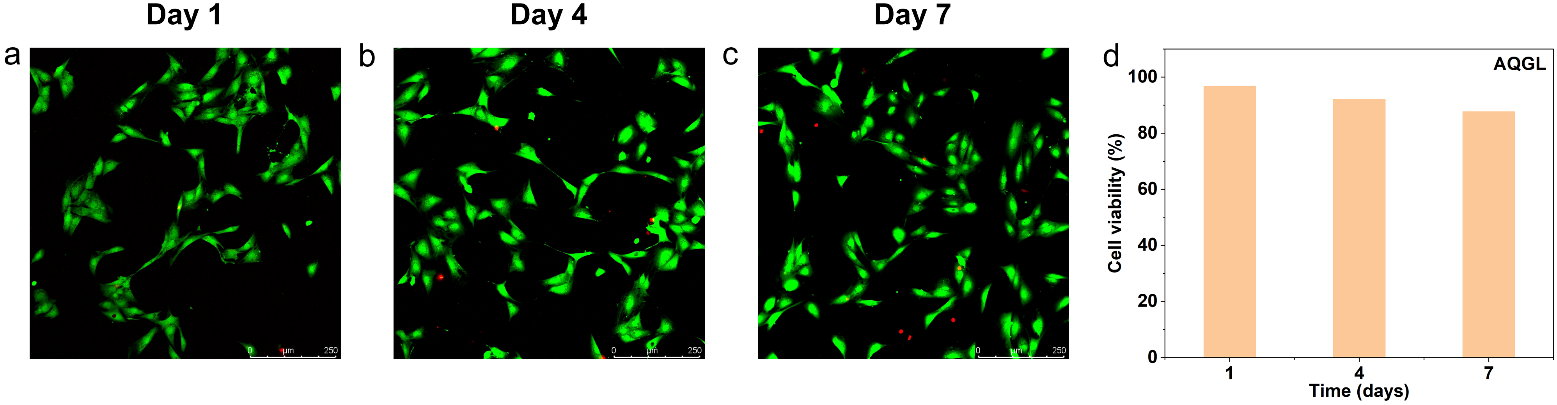


Figure S18. Confocal fluorescence microscopy image of the MC3T3-L1 cells cultured on the AQGL gel for (a) 1, (b) 4, and (c) 7 days. (d) Cell viability of the cells cultured on AQGL gel at 1, 4, 7 days.


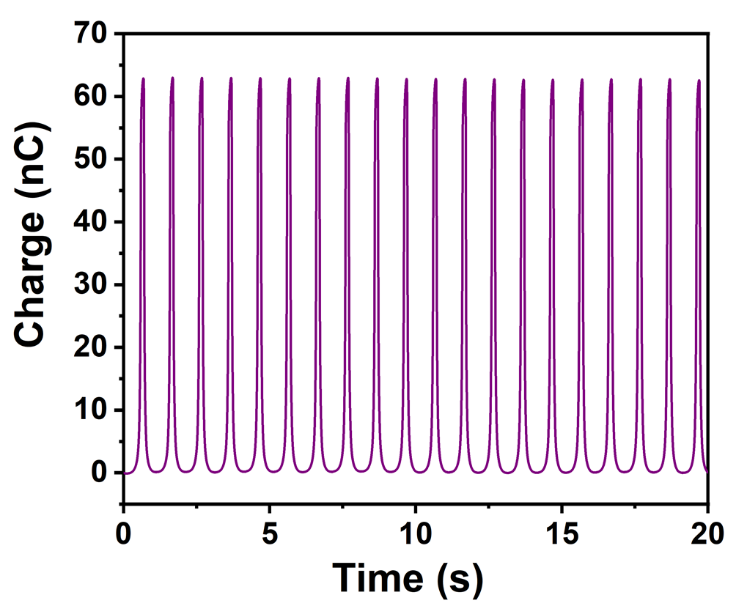


Figure S19. Transferred charge of the AQGL-TENG driven by a fixed mechanical force of 10 N at 1 Hz.


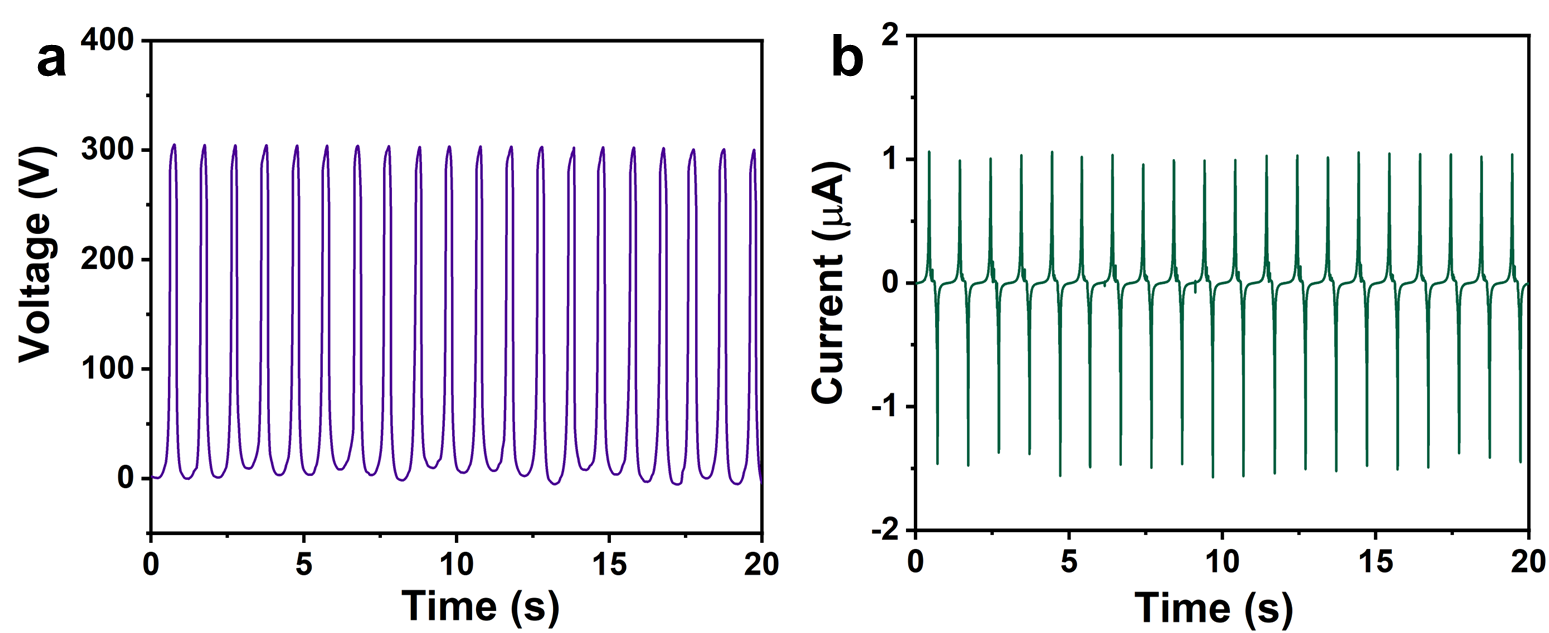


Figure S20. Generated open-circuit voltage and short-circuit current of the AQ-TENG under constant mechanical loading (10 N, 1 Hz).


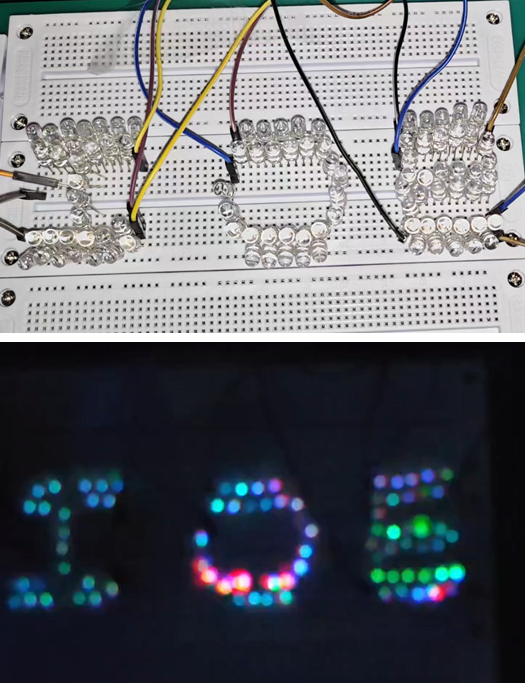


Figure S21. Instantaneous illumination of 80 LEDs powered by the AQGL-TENG.


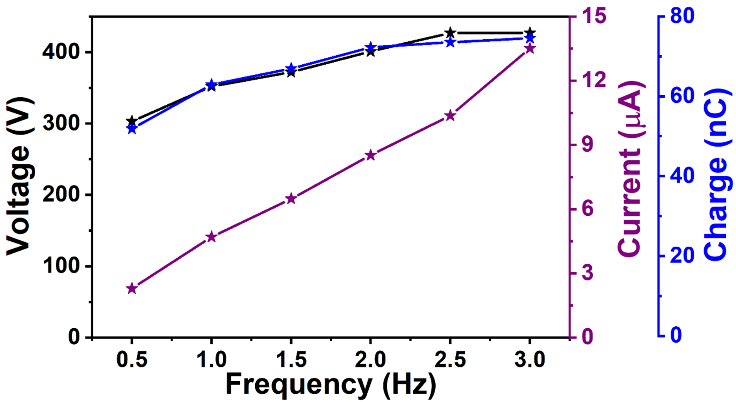


Figure S22. Variation curves of open circuit voltage, short-circuit current, and transferred charge under different driven frequencies.


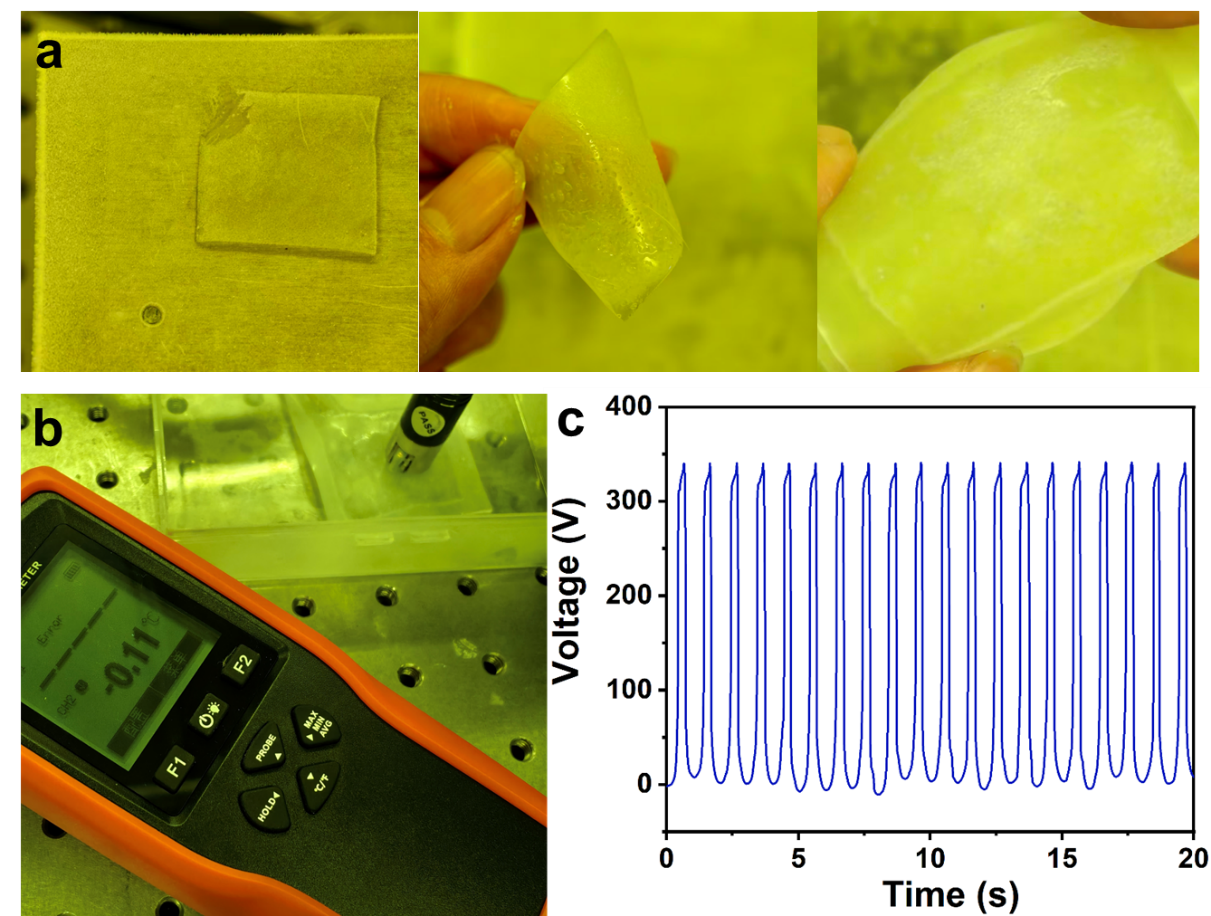


Figure S23. Output voltage of the AQGL-TENG after freeze-thaw cycles.


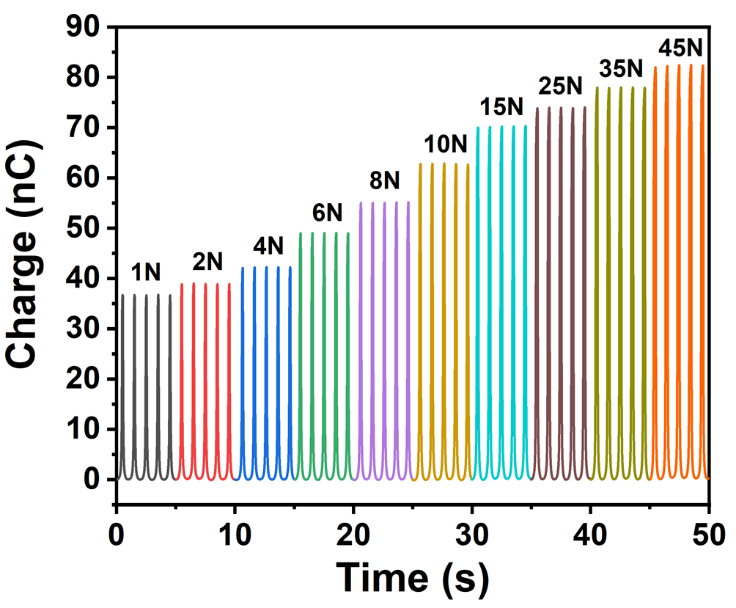


Figure S24. Effect of varying applied forces on the transferred charge.


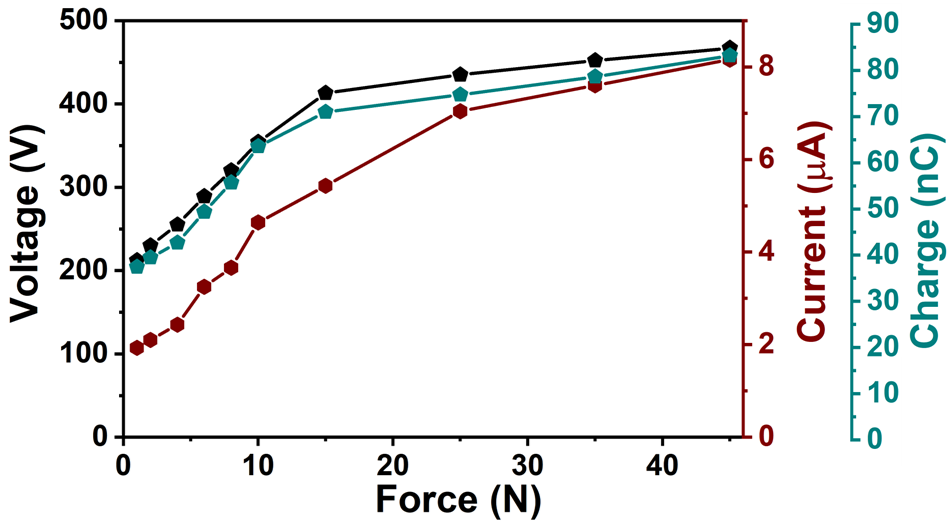


Figure S25. Variation curves of open circuit voltage, short-circuit current, and transferred charge under different driven forces.


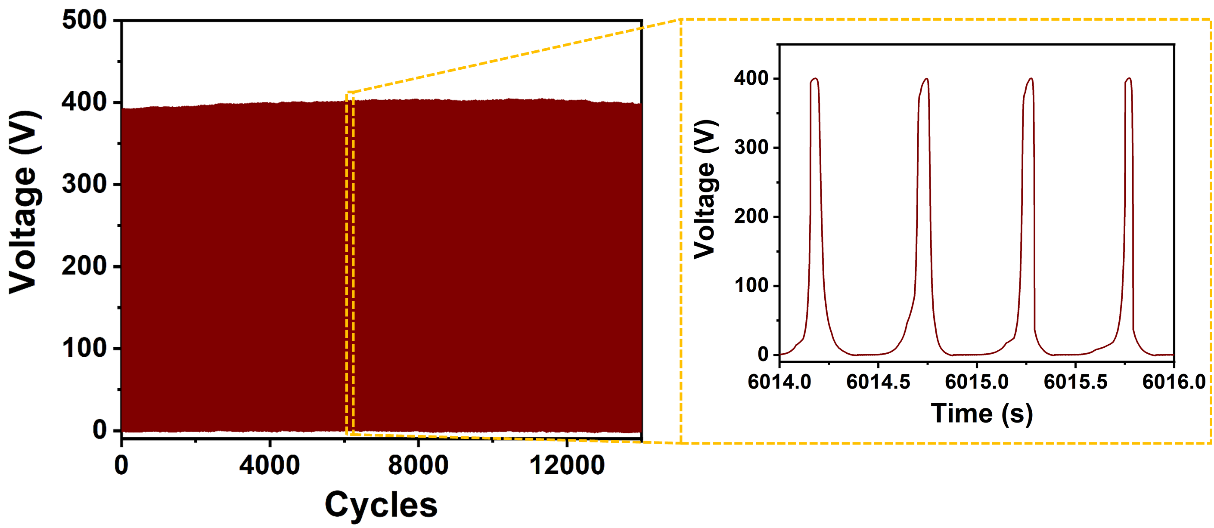


Figure S26. Mechanical stability of the AQGL-TENG evaluated over 14000 working cycles at a constant driving frequency of 2 Hz.


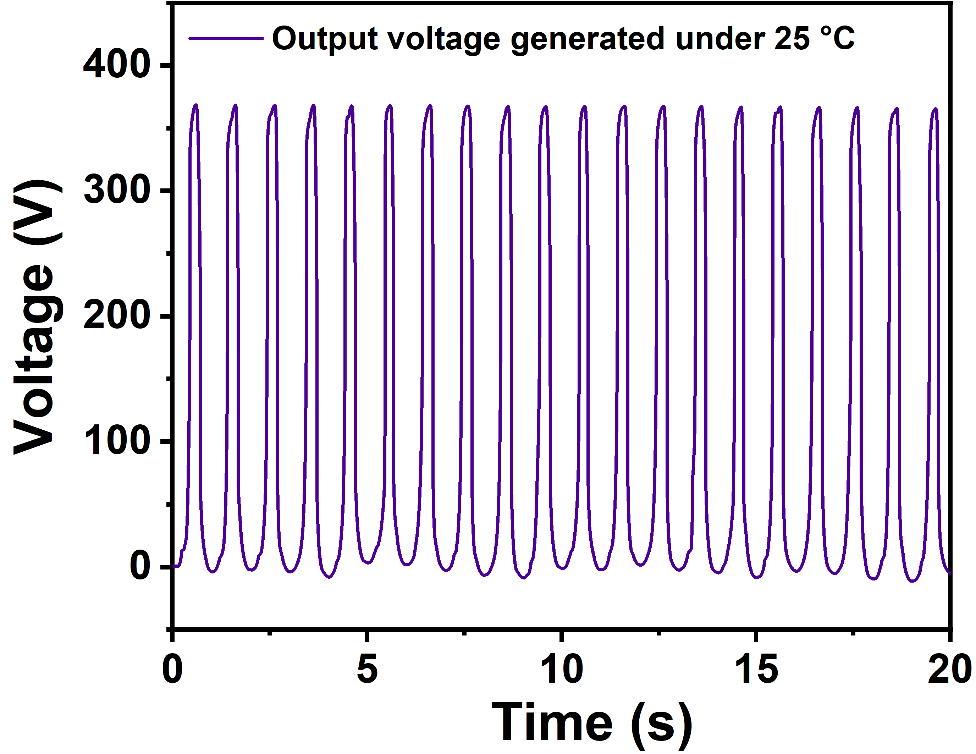


Figure S27. Output voltage of the AQGL-TENG after 100 days.


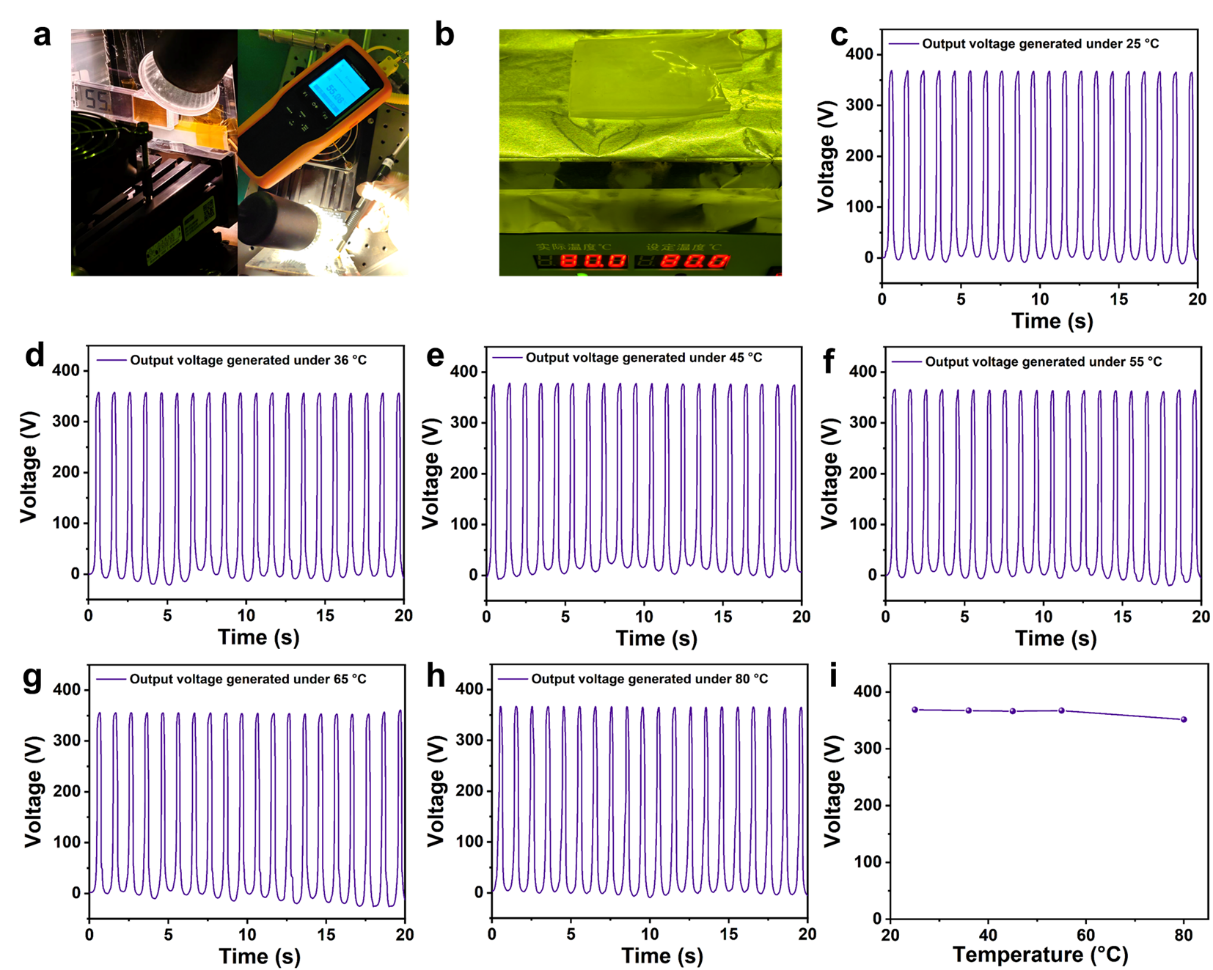


Figure S28. Output voltage of the AQGL-TENG under different temperature conditions.


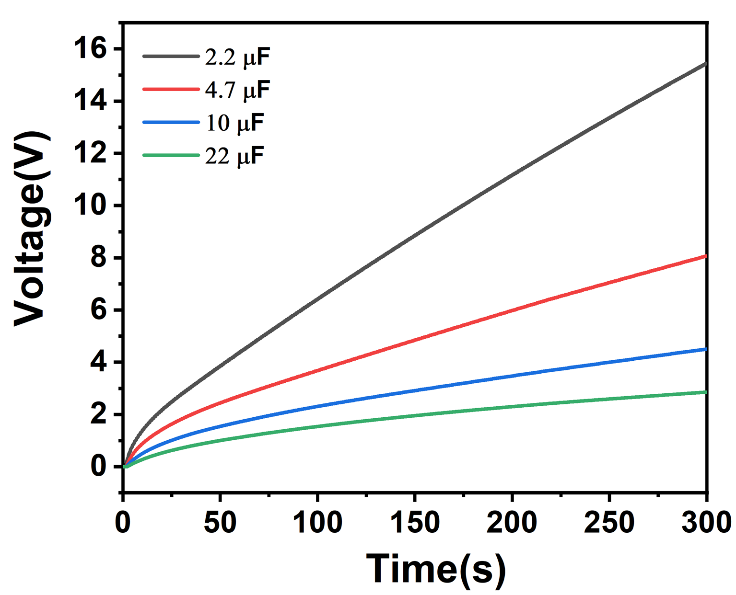


Figure S29. Charging behaviors of the AQGL-TENG toward different capacitors driven by a fixed mechanical force of 10 N at 1 Hz.


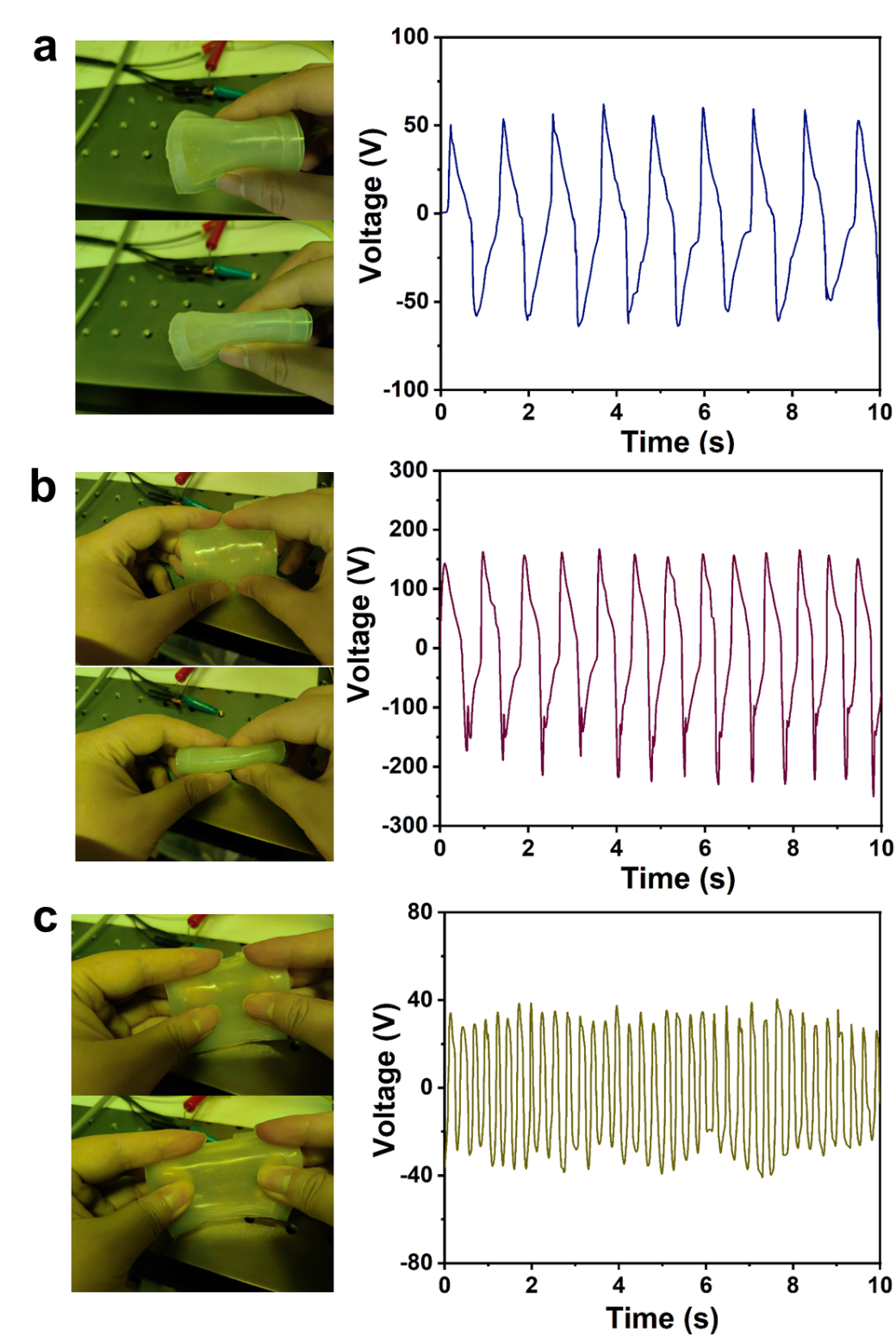


Figure S30. Output voltage of the AQGL-TENG under (a) one-hand bending-releasing cycles, (b) two-hand bending-releasing cycles and (c) stretching-releasing cycles.

**S4. Supplemental Videos**

**Video S1:** Tensile stress-strain test of the sample.

**Video S2:** Tensile stress-strain test of the notched sample.

**Video S3:** Instantaneous illumination of 80 LEDs.

**Video S4:** Charging capacitor by the AQGL-TENG.

**Video S5:** Output voltage generated from fast walking.

**Video S6:** Output voltage generated from keyboard clicking.

**Video S7:** Recording of handwriting “Y”.
